# Evaluation of the Performance of the Turkish Regulatory Agency: Recommendations for Improved Patients’ Access to Medicines

**DOI:** 10.3389/fphar.2019.01557

**Published:** 2020-02-03

**Authors:** Oguzhan Koyuncu, Hakki Gursoz, Ali Alkan, Hacer Coskun Cetintas, Tuncay Pasaoglu, Emel Mashaki Ceyhan, Stuart Walker

**Affiliations:** ^1^ Turkish Medicines and Medical Devices Agency, Ankara, Turkey; ^2^ Faculty of Pharmacy, Yeditepe University, Ankara, Turkey; ^3^ Centre for Innovation in Regulatory Science, London, United Kingdom; ^4^ Department of Clinical and Pharmaceutical Sciences, School of Life and Medical Sciences, University of Hertfordshire, Hatfield, United Kingdom

**Keywords:** Türkiye İlaç ve Tıbbi Cihaz Kurumu, good review practice (GRevP), marketing authorization, Centre for Innovation in Regulatory Science, Turkish Medicines and Medical Devices Agency

## Abstract

**Background:**

This study was to evaluate the Turkish regulatory review process and timelines between 2016 and 2018 with a view to assess the changes that had taken place since the previous study, which evaluated the Turkish review processes and timelines 2013 to 2015.

**Methods:**

Data related to the Turkish Medicines and Medical Devices Agency (TİTCK) organizational structure and general information were collected from publicly available sources. A standard questionnaire was then used to collect data with the aim of identifying the TİTCK’s review practices and key milestones for the marketing authorization process. Subsequently, a comparison with the previous study was conducted to identify the key changes and developments that had taken place from 2015- 2018.

**Results:**

The TİTCK has made considerable efforts to improve its regulatory capacity since 2016, which has contributed to the overall decrease in the agency review times. The overall median approval time for new active substances; however, increased from 529 calendar days (2016) to 663 calendar days (2018), with the review time in the agency decreasing from 408 calendar days to 326 calendar days, while the company time increased from 137 to 268 calendar days, respectively, over this period.

**Conclusions:**

For the TİTCK to become an international reference agency, they will need to fully implement good review practices and a structured framework for benefit-risk assessment and decision making; consider implementing verification and abridged reviews based on a reliance model and expedite the PIC/S mutual recognition process as well as become a full member of the *International Conference on Harmonization* of Technical Requirements for Registration of Pharmaceuticals for Human Use (*ICH)*.

## Introduction

The promotion of public health by ensuring patients’ timely access to safe, high-quality, and effective medicines is one of the most essential roles for governments and the regulation of those medicines is the primary responsibility of national regulatory authorities (NRAs). Ideally, in view of the global and dynamic environment of the pharmaceutical industry, NRAs should not work in isolation, as they are expected to ensure that their review process and related local regulations and guidelines are in line with international standards. Therefore, NRAs often work in collaboration with key stakeholders, such as the pharmaceutical industry, related disease and patients’ associations, and other NRAs (Bujar et al., 2017).

Only 26% of World Health Organization (WHO) member countries have regulatory capacity at a mature level according to WHO criteria, while the remaining 74% have not yet achieved a minimal maturity standard (Khadem, 2018). Increasing regulatory capacity is a major priority for those NRAs that have a vision to become a regional reference agency (Mashaki, 2017). The Turkish Medicines and Medical Devices Agency, Türkiye İlaç ve Tıbbi Cihaz Kurumu (TİTCK) has an aspiration to become a recognized reference agency and, therefore, has initiated a number of international projects to improve its regulatory capacity and competencies in collaboration with key international organizations, such as the WHO, the International Council for Harmonization of Technical Requirements for Pharmaceuticals for Human Use (ICH), and the Pharmaceutical Inspections and Cooperation Scheme (PIC/s). Thus, in 2018, the TİTCK became a member of PIC/s and an ICH observer member and is currently engaged in the necessary processes for becoming a full ICH member and a WHO-listed reference authority.

The purpose of this study was to evaluate the Turkish regulatory review process and timelines between 2016 and 2018 with a view to assess the changes that had taken place since the previous study, which evaluated the Turkish review processes and timelines over the 3-year period 2013 to 2015 (Mashaki, 2017).

### Study Objectives

The main objectives of this study were to:

- Evaluate the current regulatory review process in Turkey in terms of timelines and quality measures implemented by the TİTCK.
- Evaluate the current status of good review practices (GRevP) and decision-making processes within the TİTCK.
- Identify the major improvements and changes in the review system in Turkey between 2016 and 2018 in comparison with a similar study covering the period (2013–2015).
- Identify the key areas of improvements, strategic needs, and recommendations for an improved regulatory system, which would facilitate patients’ access to innovative medicines in Turkey.

## Materials and Methods

### Data Collection Process

Data related to the TİTCK organizational structure and general information were collected from publicly available sources. A standard questionnaire was then used to collect data with the aim of identifying the TİTCK’s review practices and key milestones for the marketing authorization process. The responses were reviewed and validated in face-to-face interviews by key TİTCK staff, including the Head of the Marketing Authorization Department as well as the Head of the Agency. Subsequently, a comparison of the data with the previous study was conducted in order to identify the key changes and developments that had taken place over the six-year period (2013–2018).

The number of applications and submission and approval dates from January 2016 to December 2018 were obtained for new active substance (NAS) and generic submissions. The data included information on other categories such as therapeutic area, priority classification, brand name, generic name, company name, approval date, application type, and total time in the agency and in the company. Data related to major line extension applications were not available and not included in the study.

The questionnaire was divided into five sections (see **supplement**):

- Part 1: Organization of the agency, which aimed to provide current information on the TİTCK structure, organization, and resources;
- Part 2: Types of review models, which explored the review model(s) for the scientific assessment of medicines in terms of the extent to which data were assessed in detail by the agency and how the agency might rely on the results of assessments and reviews carried out elsewhere;
- Part 3: Key milestones in the review process, which provided a process map including milestones, that had been developed from studying procedures followed in “established” and “emerging” regulatory agencies. It captured the main steps in the review and approval process and identified key “milestone” dates;
- Part 4: GRevP, which examined the key elements of the GRevP that contribute to those measures that had been adopted to improve consistency, transparency, timeliness, and competency in the review processes; and
- Part 5: Quality decision-making processes, which aimed at understanding the quality of the decision-making process within the agency and whether the agency had measures in place to ensure quality decision-making practices.

This study identified the general organizational structure of the agency including, the relationship between the TİTCK internal and external reviewers as well as the key milestones and procedures of the regulatory review process. Data were then transferred into a report, which provided a comprehensive process map and, therefore, facilitated the evaluation of the Turkish review system and its timelines. The study design included a final step to identify the key areas of improvements and changes achieved within the TİTCK since the previous study (2013–2015).

### Data Analysis

Statistical analysis of the data was conducted by using MedCalc^®^ software (Version 19). Since data did not show a normal distribution, Mann Whitney test was used to compare data on different use. Type 1 error (α) was accepted as 0.05. All tables and graphs were prepared by using Microsoft^®^ Excel for Mac 2019 (Version 16.26).

## Results

The results of this study are presented in five parts: organizational structure of the TİTCK; TİTCK review model, TİTCK regulatory review process map, milestones, and timelines; good review practices in the registration process; and quality of decision-making process.

### Organizational Structure of the TİTCK

Established in 1946, the Pharmaceutical and Pharmacy General Directorate was the official body for marketing authorization of pharmaceuticals in Turkey. In 2011, as part of Turkey’s Transformation Program, this agency was replaced by the Turkish Medicines and Medical Devices Agency TİTCK, affiliated with the Ministry of Health, and also charged with the regulation of cosmetics. TİTCK responsibilities include marketing authorizations/product licenses, clinical trial authorizations, post-marketing surveillance, regulation of advertising, laboratory analysis of samples, price regulation, and good manufacturing practice (GMP) inspections.

According to the official “Annual Activity Report 2018” issued by the TİTCK in 2019, the total number of agency staff in 2018 was 1040, 182 of whom were working as reviewers, with 64% being pharmacists (TİTCK, 2019). The agency budget in 2018 was 28 million USD, 22% of which was funded by the government, whereas the rest was from the fees allocated and paid by applicants for marketing authorizations and post-marketing variations (TİTCK, 2019).

### TİTCK Review Model

The scientific review within the regulatory review process used by NRAs can be classified as one of three types according to the level of data assessment conducted; that is, type 1 – verification review; type 2—abridged review; or type 3—full review. This classification was defined at the Centre for Innovation in Regulatory Science (CIRS) Workshop, “The Emerging Markets: Regulatory issues and the impact on patients’ access to medicines,” organized in Geneva, Switzerland in March 2006 (McAuslane et al., 2009).

The type 1—verification review model is used by a number of health authorities that lack sufficient resources and capacity to perform a comprehensive scientific review of a new marketing authorization application (MAA). This model helps reduce duplication of efforts by agreeing that the approving authority will issue a marketing authorization for any product once the product is officially approved by two or more recognized reference countries. The reference countries will be those that the agency trusts in terms of their review and with whom they may have a memorandum of understanding. The main responsibility of the local authority is to ensure the “verification” of all data submitted as declared in the application dossier. This includes the verification review of the product characteristics (formulation, composition, and strength) and the proposed labeling information (use, dosage, precautions) for local marketing and that it complies with the reference country(s) authorization(s). Approval evidence from recognized reference countries, such as the submission of Certificate of Pharmaceutical Product (CPP), is a pre-requisite for such applications in this review model.

The type 2—abridged review model ensures the optimal use of the available resources by the local authority by not re-assessing the scientific supporting data included in the MAA, as long as these data have been evaluated and approved by one or more of the recognized reference country authorities. However, the MAA still undergoes an abridged review in relation to the product’s use and characteristics in the local market. Therefore, the abridged review model usually contains confirmation of the scientific clinical data, but also includes a local review of quality data or chemistry, manufacturing, and controls (CMC) of the product. The review of the quality data is mainly to confirm the product’s stability in relation to climatic conditions and distribution infrastructure in the local country. Moreover, the local review of clinical data might include a benefit-risk assessment in relation to its use in the local ethnic population, medical practice/culture, and patterns of disease and nutrition in the country. In the abridged review model, approval by a recognized agency elsewhere is a pre-requisite before the local authorization can be granted, but the initial application need not necessarily be delayed until formal documentation such as a CPP is available, but this must be provided before final authorization.

In the type 3—full review model, the authority has suitable resources and capacity to perform a full independent scientific review. This includes collaborating with both internal and external experts to carry out a full review and evaluation of the supporting scientific data (quality, pre-clinical, clinical) for a major application. Full review models do not require a marketing application approval in any other country at the time of the submission and thus can be carried out earlier or in parallel with review of first applications worldwide. However, in some countries, local regulation requires an evidence of approval in the country of origin or a reference agency prior to local approval being granted.

#### Data Requirements and Assessment

According to the Turkish “Law on Pharmaceuticals and Medical Preparations” and the “Regulation on Marketing Authorization of Human Medicinal Products,” pharmaceutical products must obtain an MAA by the related regulatory authority to be marketed (TİTCK, 2005). In order to obtain approval, the applicant needs to meet all the following criteria: the applicant needs to be a legal entity in Turkey, the documents that are listed under the Marketing Authorization Regulation should be submitted to the agency, the submission should be performed electronically *via* the official system.

The submission dossier format should be in compliance with the ICH Common Technical Document (CTD) format, which consists of the five modules including quality, efficacy, and safety information about the product. Even though the proof of approval such as the CPP is not a requirement at the time of submission, for final approval the applicant should submit the approval documents (TİTCK, 2005).

### TİTCK Regulatory Review Process Map, Milestones, and Timelines

**Figure 1:**
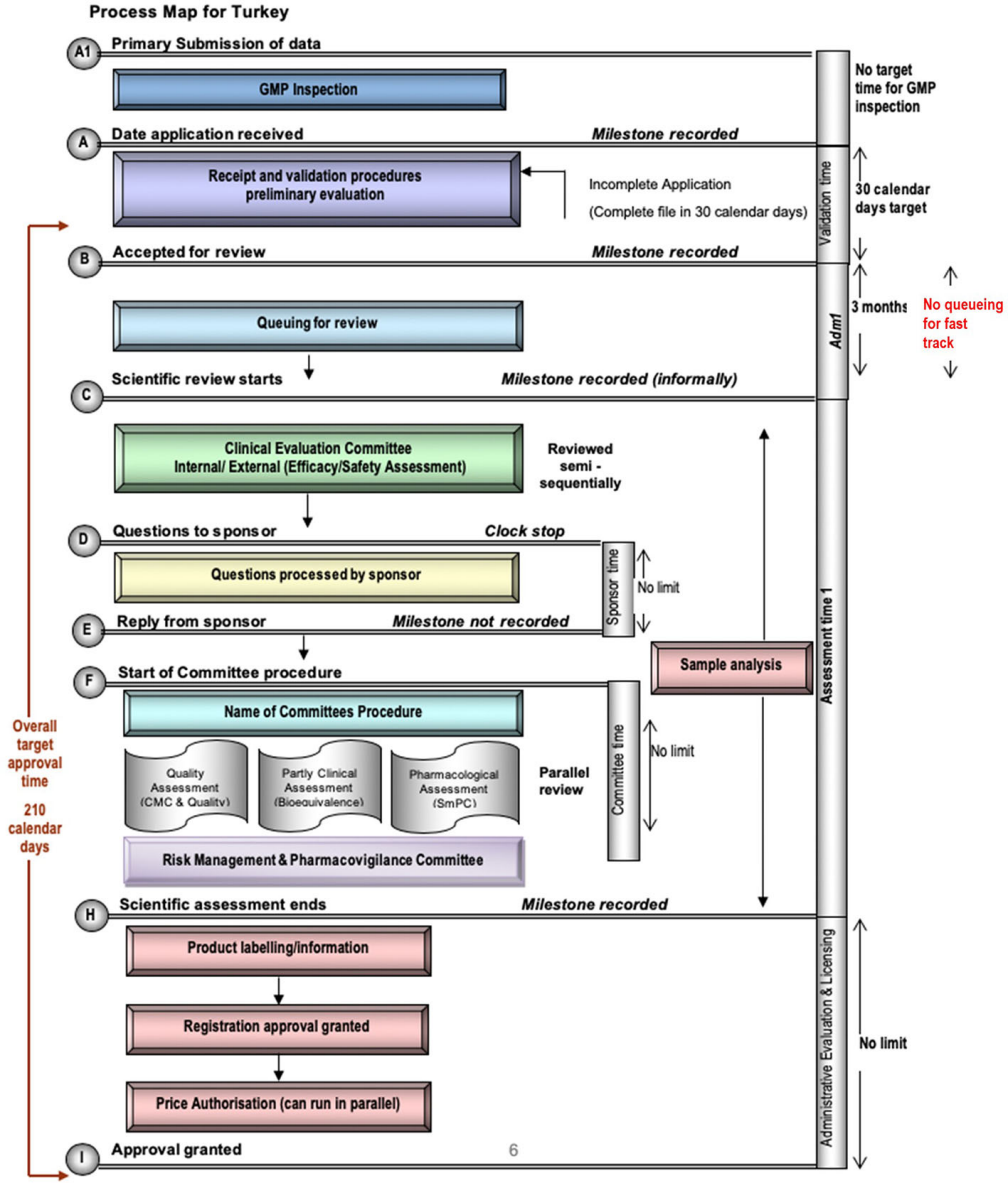
Process Map for TİTCK.

Currently the TİTCK only conducts a full review of NASs. A map of the review process in Turkey is illustrated in **Figure 1**, which identifies the TİTCK registration review process from the first step, which is the validation (also referred to as the preliminary review) of the application to the last step, which is the final approval. Moreover, the process map highlights the key milestones and standardized registration review process, which facilitates comparison of TİTCK with other regulatory authorities. The pre-submission steps for GMP accreditation or prioritization of the submission and the steps or processes related to rejection and deficiencies are not included.

GMP accreditation for the manufacturing site of the product must be completed before marketing authorization application. Exceptionally, the application for critical products, such as those for unmet medical needs or orphan drugs, can be submitted and reviewed in parallel to an ongoing GMP accreditation process to save time. To obtain GMP accreditation, a GMP dossier that includes manufacturing and quality data related to the manufacturing site or line must be submitted by the applicant. Following dossier approval, the applicant is informed about the possible inspection date and scope. If the manufacturing site has been previously inspected, according to the result of the risk-based approach, the inspection may be carried out paper-based instead of an on-site inspection.

The primary milestone and official commencement of the registration process is the validation of the dossier, which is submitted electronically *via* the TİTCK system. In this milestone, the submitted dossier is reviewed in terms of format and content. The dossier needs to be in compliance with the CTD format and include related modules depending on whether the application type is a new drug or a drug that requires an abbreviated format such as a generic, hybrid, or biosimilar. Related scientific data, administrative documents such as the legal status of the applicant, GMP certificate, and payment of fees should be included in the appropriate modules. A CPP is requested at this time, with no restriction regarding recognized authority.

The validation of the dossier has to be completed in thirty calendar days according to the regulation. In the case of deficiencies, the applicant has an additional thirty days to respond. Following the response of the applicant, the TİTCK has to complete the review again in thirty days. If deficiencies still exist, the TİTCK has to reject the dossier. If the applicant decides to make a new application, the process starts over again with the payment. Upon completion of the validation step, the application is placed in a queue to be forwarded for the scientific review phase, which must occur within a maximum of three months, except for prioritized applications, which bypass queueing. This mechanism is based on the management of applications according to workload in the registration review process. This is achieved in the planned period by limiting the number of applications in the assessment process, allowing applications to be managed by the agency despite any potential backlogs.

#### Scientific Review Process

The TİTCK always performs a full review and the submitted dossier is assessed scientifically in terms of efficacy, safety, and quality. The scientific assessment is carried out by several scientific committees, which consist of academicians, scientists, and internal agency experts. The first step of the scientific assessment is the clinical evaluation of the submitted data by the Clinical Evaluation Committee. With its overall scientific assessment, this committee answers the question “Do we as patients and health professionals need this product?” establishing the rationality of the marketing authorization of the product. Expert committees conduct scientific reviews.

#### Expert Committees

There are thirty committees with a total of three hundred members who are responsible for the scientific assessments of the products including medicines, medical devices, and cosmetics. The committees consist of internal experts among the agency staff and external experts from the universities around the country. The required skills for committee members are detailed in the legislation “Regulation on the Establishment and Roles of the Turkish Medicines and Medical Devices Agency’s Scientific Advisory Committees,*”* but there are no written rules that specify the selection methods for these experts. The experts, who are elected for three-year terms, are mandated to sign confidentiality and conflict of interest agreements with the TİTCK. The committees meet weekly with the agenda to scientifically review submitted data prepared by the agency staff, but there is no target time for the scientific assessment by the committees.

#### Scientific Assessment Sequence

The sequence of the scientific assessment phase depends on the application type and also the approval status of the product by other authorities such as European Medicines Agency (EMA) and US Food and Drug Administration (FDA). For a product that has already been approved/registered by any authority, the registration review process starts with the assessment by the Clinical Evaluation Committee. Following the committee’s positive decision, the submission is then reviewed by the Bioavailability and Bioequivalence Committee, Pharmacological Evaluation Committee, and Quality Evaluation Committee, in parallel. For a product that is not approved but, is being simultaneously assessed by another authority, the review process commences with the assessment by the Bioavailability and Bioequivalence Committee in parallel with the Quality Evaluation Committee. The next steps are the assessment of the Clinical Evaluation Committee and the Pharmacological Evaluation Committee. The quality control analysis by the national laboratory, the pharmacovigilance review, which is a part of the benefit-risk assessment, and the pricing process, which must be completed before the marketing authorization, can be conducted in parallel and independently to the review process. Questions and queries can be raised related to the application by the experts throughout the review process at any time. These questions are sent to the applicant separately, not as a batch, and there is no official time limit for the company to respond. Occasionally, the deadline for the response is stated in the official letter. But the time for the company response (clock stop) is not included in the total assessment time for the product.

#### Interaction of TİTCK With the Applicants

TİTCK does not provide pre-application scientific advice for companies, unlike the EMA and FDA and other mature regulatory agencies; however, written and, in special cases, verbal discussion of the application with the TİTCK is possible during the review period, and there are no fees for applicant-arranged TİTCK meetings. Exceptionally, meetings with an expert committee may be arranged for the company to make an oral presentation in order to discuss key issues regarding specific and scientific questions.

#### Prioritization of the Application

In addition to the abbreviated application with a shorter pathway for generics and biosimilars, the TİTCK classifies an application for a critical product as either priority or high priority. This classification provides a priority in the regulatory review process and an accelerated MAA for the product. To secure this privileged designation, the product should be the first generic in a class, a biosimilar, an innovative product in terms of public incentive, a strategic product in terms of the state policies, or a vaccine. The designation of the product as high priority or priority is made by the Prioritization Committee, which consists of a number of experts, key officers, and several high-level managers of the TİTCK: Vice President of the Medicines and Pharmacy, Vice President of the Economic Assessments and Laboratory Services, Head of the Marketing Authorization Department, Head of the Economic Assessments and Medicines Supply Management Department, Head of the Analysis and Control Laboratories, Head of the Medicine Inspection Department, and Head of the Medicines and Pharmacy Department from the Social Security Agency.

#### Pricing and Its Methodology

The pricing procedure in Turkey is completely independent of the marketing authorization process. However, after the marketing authorization and to obtain sales permission, the official price of the product must be determined by the agency, using the reference pricing system. According to this system, the lowest price for the product is selected from among the prices in five reference countries in the Mediterranean region (currently, Portugal, France, Greece, Italy, and Spain), and this price is then converted to the Turkish currency using a fixed exchange rate.

#### Decision of Approval

After the completion of the scientific review, a dedicated unit, responsible for the cross/final check of all administrative documents, expert committees’ decisions and laboratory analysis, as well as the label, evaluates the submission dossier. This is the last step before the marketing authorization, which is granted by The Head of the Turkish Medicines and Medical Devices Agency.

#### TİTCK Review Timelines

According to the Article 15 in the Turkish regulation, the time for market authorization should not exceed 210 calendar days. Therefore, target timelines for the TİTCK regulatory review system are 210 calendar days for scientific assessment and authorization of standard applications, 180 calendar days for priority applications and 150 days for high-priority applications. Priority applications and high-priority applications are determined on the basis of whether the product meets an unmet medical need, is the first generic to be marketed, or is critical to public health. These criteria are assessed using a points system with the highest points determining high priority and lower points indicating priority.

Milestone targets are 30 calendar days for validation; no target for queuing/backlog; no official target for scientific assessment, but unofficial target is 8 months; no official target for authorization process, but unofficial target is 30 days.

**Figure 2:**
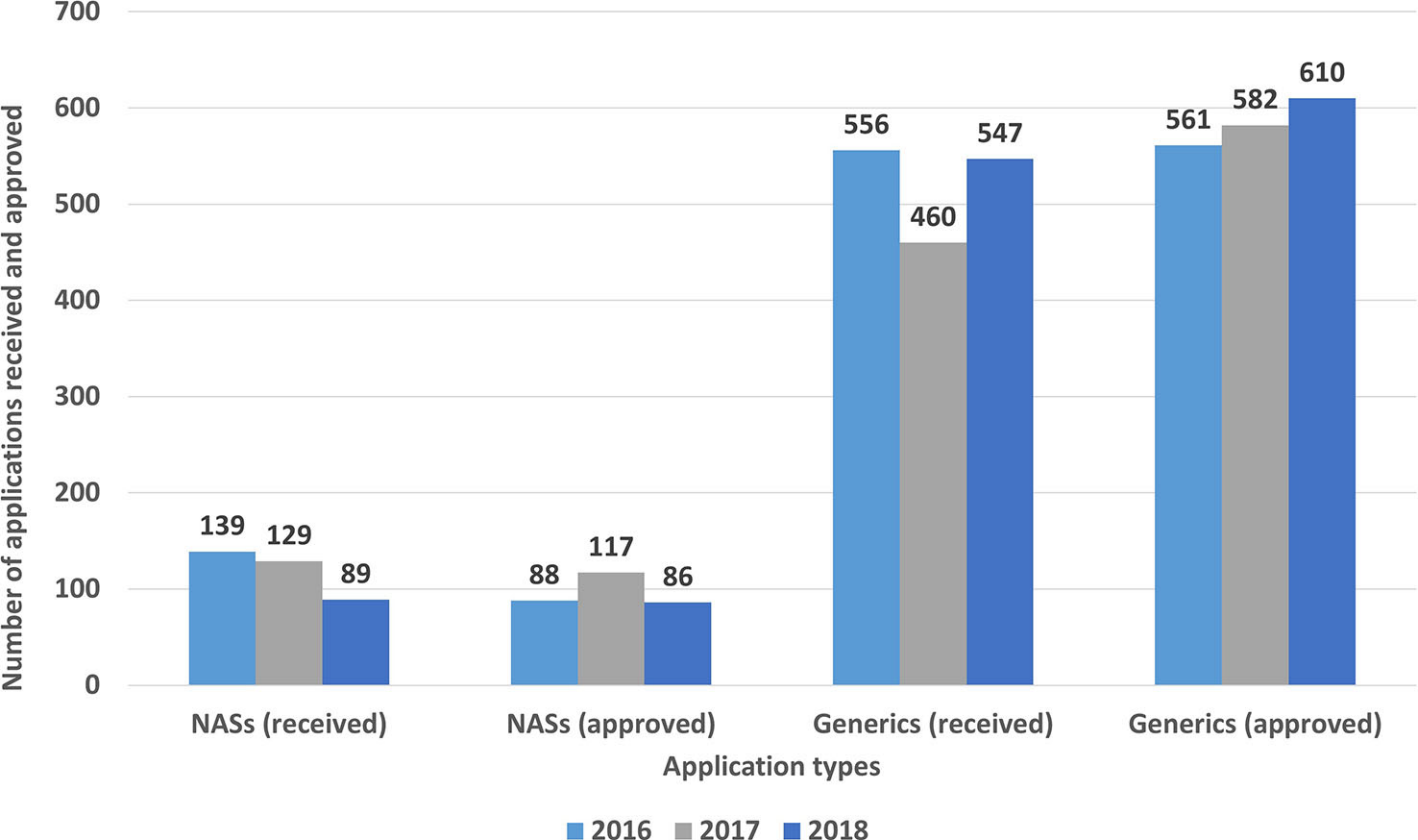
New active substance and generic applications received and approved (2016–2018).

The number of marketing authorization applications and approvals, which varies depending on the type of product (NASs and generics), is presented in **Figure 2**. The number of NASs approved ranged from 86 (2018) to 117 (2017), whereas the number of generic applications approved ranged from 561(2016) to 610 (2018). Furthermore, the NASs were classified according to the Anatomical Therapeutic Chemical (ATC) classification system which is recommended for drug utilization studies by WHO (**Table 1**). The top five ATC approvals for 2016, 2017, and 2018 were, respectively, antineoplastic and immuno-modulating agents, alimentary tract and metabolism, anti-infectives for systemic use, blood and blood-forming organs, and nervous system products.

**Table 1:** Number of new active substances approved according to ATC class.

Anatomical Therapeutic Chemical (ATC) Class	2016	2017	2018	Total
Alimentary Tract and Metabolism	7	13	24	44
Anti-infectives for Systemic Use	16	15	7	38
Antineoplastic and Immuno-modulating Agents	28	37	25	90
Blood and Blood Forming Organs	7	12	9	28
Cardiovascular System	12	1	1	14
Dermatological	2	0	0	2
Genito Urinary System and Sex Hormones	4	5	9	18
Nervous System	8	15	2	25
Respiratory System	1	2	2	5
Sensory Organs	0	1	1	2
Systemic Hormonal Prep., Excl. Sex Horm. And Insulins	0	11	1	12
Various	3	5	5	13
Total	88	117	86	291

The approval numbers were categorized according to priority designation. In the first year, the total number of approved applications granted priority was only 5 (4% of all NAS applications) whereas 8 products were designated as high priority (7% of all NAS applications). However, in 2018, when prioritization was embedded by all stakeholders with the increase in the recognition of the prioritization process, the number of priority approvals as well as high-priority applications increased to 35 (41%).

**Figure 3:**
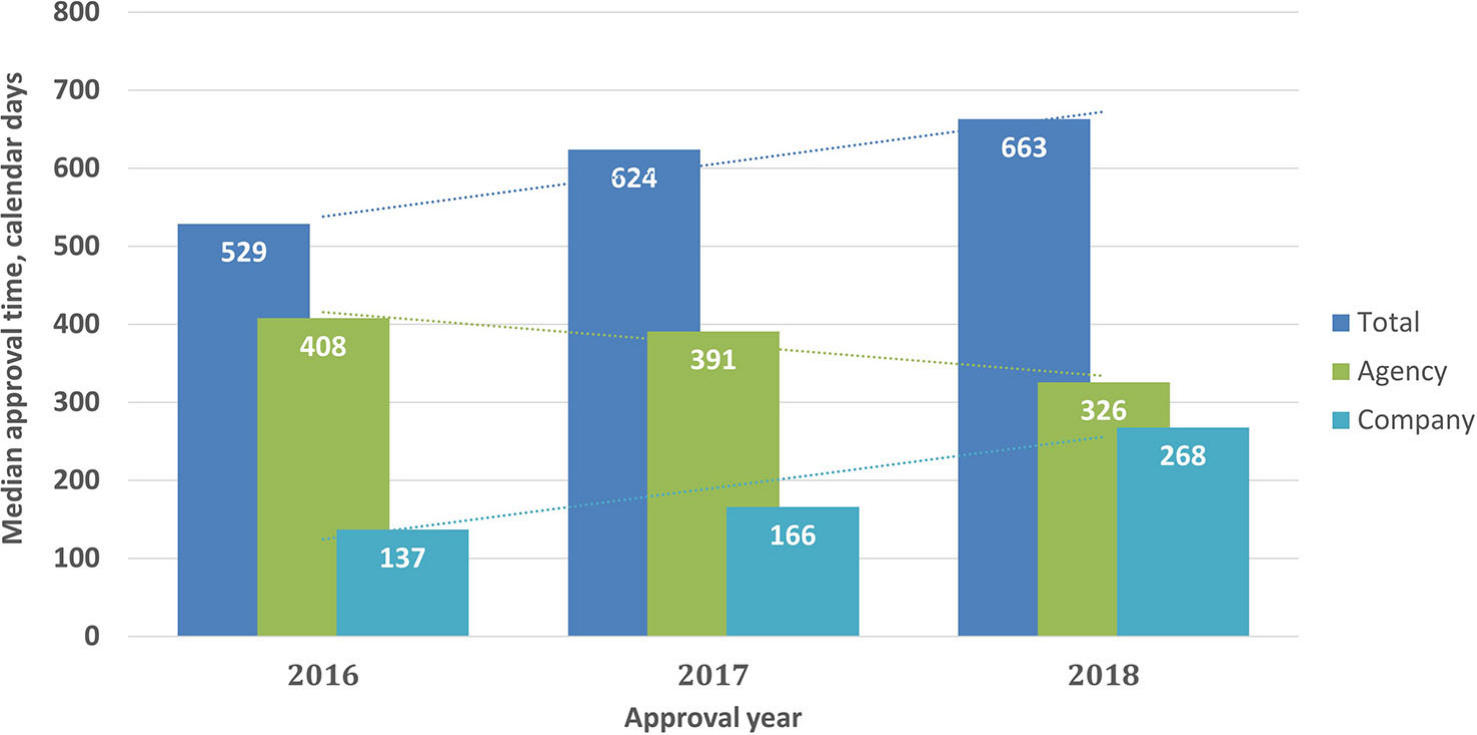
Median approval times for new active substances (2016–2018) Validation time was not included.

Approval timelines are a major indicator of regulatory performance. For NASs (2016–2018) the results showed that the median approval times (from validation to final approval) were 529, 624, and 663 calendar days for 2016, 2017, and 2018, respectively, including both agency and company response time. However, this increase was not statistically significant (P = 0.34). Agency response time was reduced from 408 days in 2016 to 326 days in 2018, whereas company time increased from 137 days in 2016 to 268 days in 2018. These changes were statistically significant (P < 0.001) (**Figure 3**).

**Figure 4:**
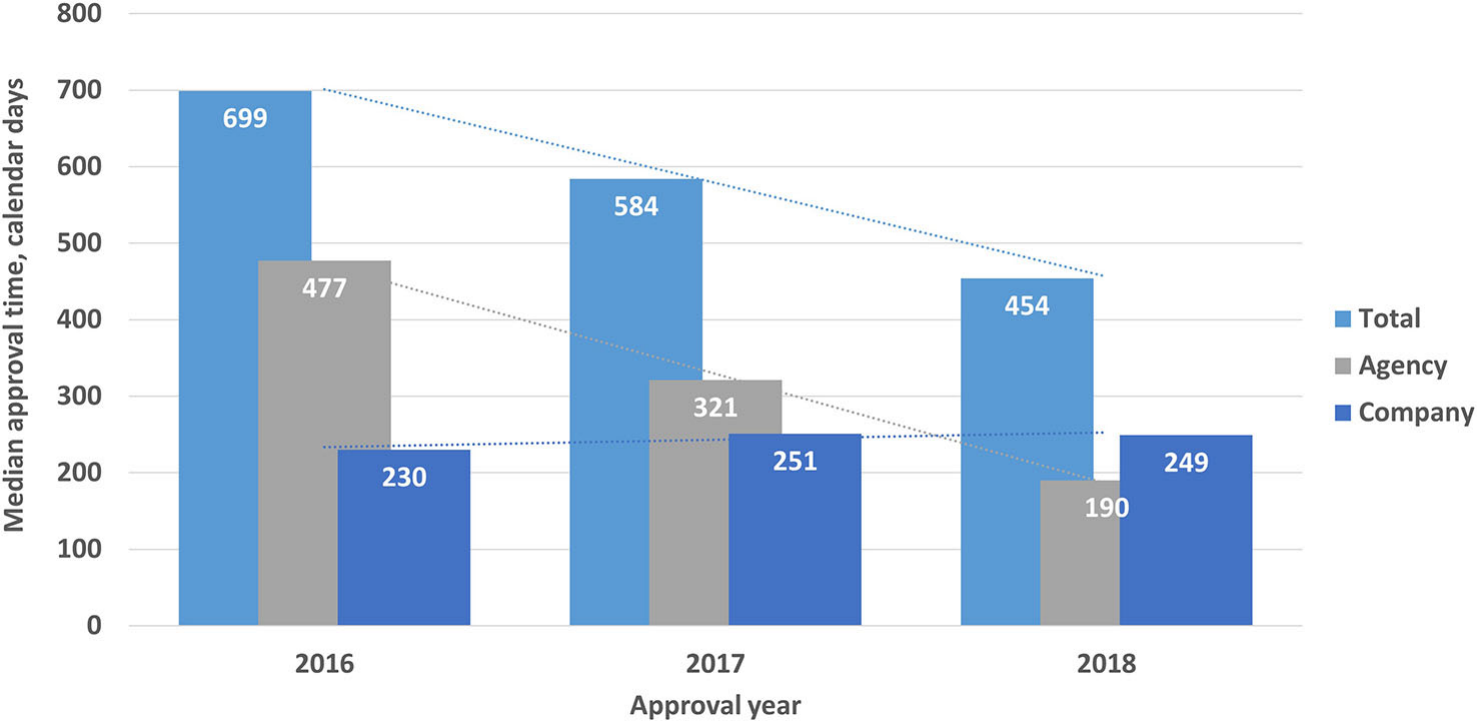
Total median approval, times (agency and company) for generics (2016–2018). Validation time was not included.

For the generics, the total approval timelines were 699, 584, and 454 calendar days for 2016, 2017, and 2018 respectively. Again, the agency time decreased from 477 days in 2016 to 190 days in 2018, whereas the company time increased from 230 days in 2016 to 249 days in 2018. All these changes were statistically significant (P < 0.001) (**Figure 4**).

The timelines were also evaluated according to the top five ATC classes. The total median approval times for antineoplastic and immuno-modulating agents as well as alimentary tract and metabolism products were relatively stable from 2016 to 2018 with the approval timelines ranging from 553 days to 663 days for antineoplastic and immuno-modulating agents, respectively, and from 606 days to 695 days for the alimentary tract and metabolism products, respectively. The review times for anti-infectives for systemic use ranged from 553 to 1287 days (2016–2018), for blood and blood-forming organs the range was 517 to 272 days (2016–2018) and for nervous system products the range was 500 to 727 for 2016 to 2018, respectively.

**Figure 5:**
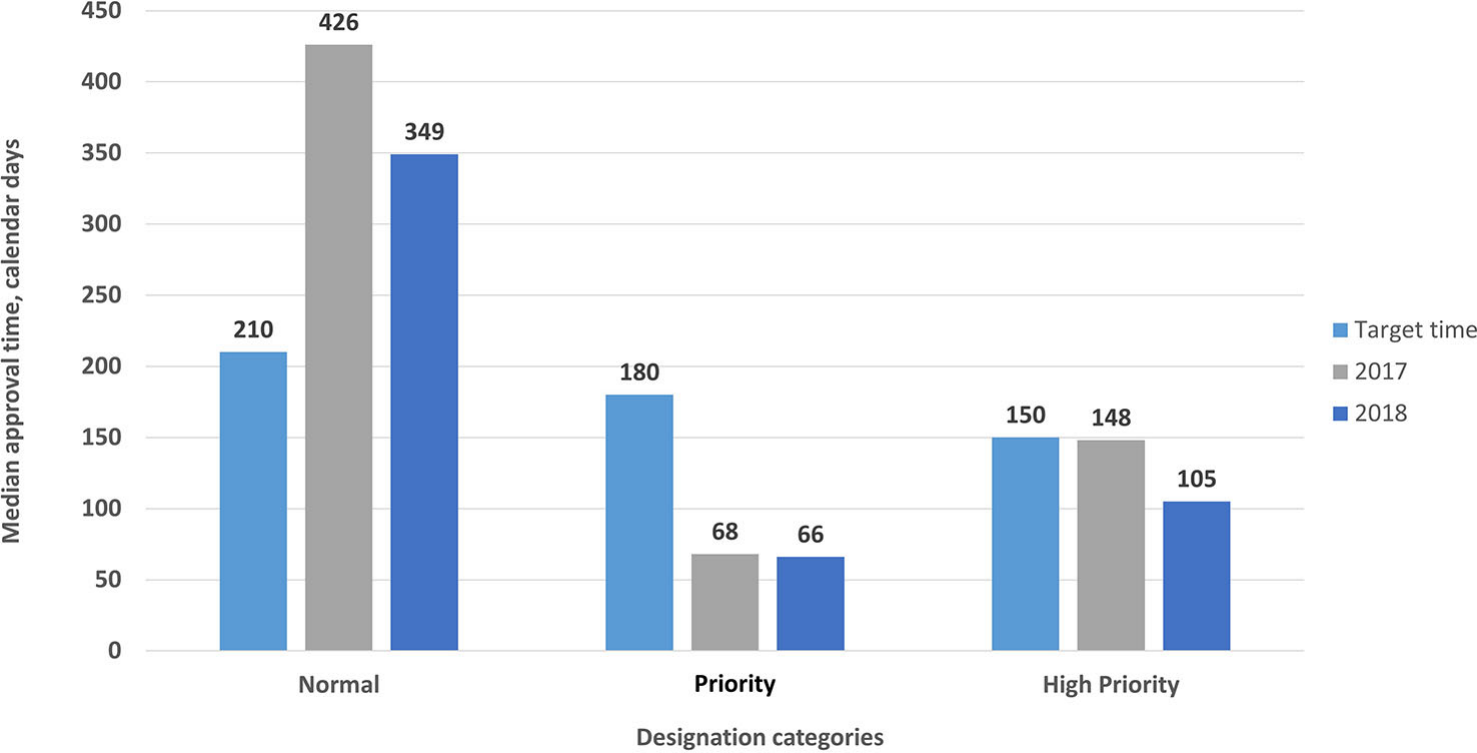
Median time for new active substances in the agency (excluding company response time) according to priority designation categories.

In addition, the median approval timelines within the agency were categorized according to the designation granted as high priority (150 days target approval time), priority (180 days target approval time) and standard (210 days target approval time). Accordingly, the timelines for high priority and priority applications were less than the target approval times for both 2017 and 2018 (**Figure 5**).

### GRevP in the Registration Process

Quality in the assessment and registration process is important for regulatory authorities, as it ensures consistency, transparency, timeliness, and competency in the review process. Regulatory authorities are continuously developing and implementing a variety of measures to improve and achieve higher-quality standards and to meet the expectations of industry and the general public (McAuslane et al., 2009). The purpose of the questionnaire’s fourth part was to obtain an insight into the strategies, measures, and resources that the TİTCK have in place to develop and maintain quality in their review processes.

The WHO has developed GRevP guidance to ensure quality in the review process and to improve regulatory capacity for NRAs (World Health Organization, 2014). The TİTCK has not implemented formal GRevP, but some of the GRevP elements being covered informally in various standard operating procedures (SOPs) with an internal quality policy and the agency has a plan to implement all GRevP elements by 2021. Additionally, the agency has implemented some SOPs for administrative processes related to pharmacovigilance and labeling as well as for the expert committees conducting scientific assessments. In addition, rather than having an assessment template, a number of checklists are used by the expert committees to facilitate the review process of the application.

The TİTCK does not have a structured assessment template nor does it prepare a public assessment report that summarizes the rationale and justification of their review decisions. However, there are plans to develop assessment templates by 2020. Also, the TİTCK does not have a peer review process, but the final check is carried out by internal reviewers after the completion of the scientific assessment.

#### Quality Management

The TİTCK recognizes the need to be more efficient, minimize/reduce errors, and ensure consistency to enhance agency quality processes. The TİTCK also endeavours to bring continuous improvements in the assessment and authorization processes by taking into account assessors’ and stakeholders’ feedback, including that arising from routine department meetings and workshops and subsequently acting on that feedback. Two dedicated staff from the Internal Audit Department may be designated for assessing and assuring quality in the regulatory review process. This department is directly responsible to the President, and they provide him with regular reports.

#### Quality in the Review and Assessment Process

The TİTCK does have official guidelines to assist and support industry in the registration of medicinal products. These guidelines are currently only in Turkish and are accessible through the agency website. Pre-submission scientific advice meetings implemented by a number of international regulatory health authorities are not available through the TİTCK. Nevertheless, some formal contacts and meetings can be conducted during the development and assessment phases of a product; although direct contact with related technical staff or reviewers to discuss an application is not permitted.

#### Training and Continuous Education as an Element of Quality

The TİTCK does not have a formal training programme for assessors, although it does carry out induction and on-the job training as well as provide external and in-house courses. Moreover, the agency supports staff obtaining post-graduate degrees by restructuring their working hours and encourages participation in international workshops and conferences. In addition, external speakers, either domestic or foreign, are invited for training in specific technical issues. Furthermore, the TİTCK seeks collaboration with more experienced agencies and agency staff can attend other agencies’ inspections, reviews, and training. However, the completion of training courses is not required for professional advancement.

#### Transparency and Communication

An open and transparent relationship with all stakeholders, including the public, professional organizations, and industry is designated as a high priority by the TİTCK. The main drivers for establishing transparency are political will and the need to increase confidence in the system as well as to provide assurance on safety safeguards. To ensure this transparency, the TİTCK conducts periodic high-level advisory meetings with stakeholders and circulates any draft regulations for the view and comments of stakeholders as well as the metrics of its regulatory review process in annual reports and scientific articles. The agency has also established a “Frequently Asked Questions” section for stakeholders on its official website. Information regarding the approval of products is available to the public *via* official journal and publications. Companies are able to follow the progress of their own applications through an Electronic Information Management System (EBYS) which allows them to access the status of their applications.

### Quality of Decision Making Process

While the TİTCK considers various types of information to carry out their assessment of new medicines, it is not always clear how decisions, which require human judgment and interpretation, are made around the data. A framework is a set of principles, guidelines, and tools, which provide a structured systematic approach to guide decision makers in selecting, organizing, understanding, and summarizing subjective values and judgments that form the basis of a decision, as well as communicating the evidence relevant to the decision. To date, the TİTCK has not developed or implemented such a framework, citing the lack of a validated framework as the main reason. Resource and administrative limitations are also among the reasons that a framework has not been implemented, although the TİTCK has a plan to adopt such a framework by 2020. However, the Centre for Innovation in Regulatory Science has engaged in considerable work to develop quality decision-making practices, including the creation of a framework that has been implemented by several organizations with a view to improving their decision making.(Donelan et al., 2016; Bujar et al., 2016) The current situation regarding the implementation of GRevPs within the TİTCK regulatory review system is presented in **Table 2**.

**Table 2:** Implementation level of good review practices within the TİTCK.

Indicator		Status		Comments
**Quality measures**	Internal quality policy	✓		Planned to update
	Good review practice system	✓		Planned to formally implement
	SOPs for guidance of assessors	✓		Planned to formally implement
	Assessment templates	X		Planned to formally implement
	Dedicated quality department	✓		Ad hoc assessments
	Scientific committee	✓		
	Internal peer reviews	✓		Planned to formally implement
	Shared and joint reviews	X		
**Transparency and** **communication** **parameters**	Feedback to industry on dossiers	✓		
	Details of technical staff to contact	X		Only face to face meetings
	Pre-submission scientific advice	X		Planned to formally implement
	Official guidelines to assist industry	✓		Available only in Turkish
	Tracking of progress of applications	✓		
	Summary Basis of Approval	X		Planned to formally implement
	Approval times	✓		
	Advisory committee meeting dates	✓		
	Approval of products	✓		
**Training and education**	International workshops/conferences	✓		Planned to formally implement
	External courses	✓		Planned to formally implement
	In-house courses	✓		Planned to formally implement
	On-the-job training	✓		Planned to formally implement
	Invitation of external speakers	✓		Planned to formally implement
	Induction training	✓		Planned to formally implement
	Sponsorship of postgraduate degrees	✓		Planned to formally implement
	Placements in other RA	X		Planned to formally implement
**Continuous improvement** **initiatives**	External quality audits	X		
	Internal quality audits	✓		
	Internal tracking systems	✓		
	Review of assessors’ feedback	✓		
	Reviews of stakeholders’ feedback	✓		

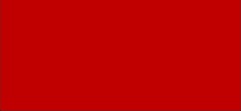
 Not implemented.

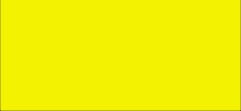
 Informally implemented.

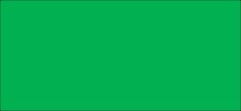
 Formally implemented.

## Discussion

Although the TİTCK was established in 2012, Turkey has had a long history and considerable experience in the regulatory arena, regulating the pharmaceutical industry since the 1920s and in issuing marketing authorizations for medicines since 1946. Recently, the TİTCK has carried out a number of key investments to improve its infrastructure as well as efforts to enhance its regulatory capacity to become an internationally recognized reference agency. For this purpose, the TİTCK initiated a number of projects with international bodies, such as the WHO and ICH, as well as centers of excellence including CIRS. Accordingly, in 2015 a study was conducted to evaluate the regulatory capacity and performance of the TİTCK for the period 2013 to 2015. This study identified possible areas of improvement within the Turkish review system and timelines and proposed a number of recommendations including a new improved review model. As an outcome of that first study, several initiatives were undertaken to address the priority areas for improvement and to identify the changes to be achieved within the TİTCK regulatory review process from 2016 to 2018. This study was initiated by conducting a detailed regulatory analysis of the TİTCK based on data collected for 2016, 2017, and 2018.

### TİTCK Organizational Structure

The TİTCK is affiliated with the Ministry of Health and the head of the agency reports to the Vice-Minister. Therefore, the TİTCK is not a fully independent agency and this often causes delays in some major decision-making activities such as the publishing of new/updated legislations. Nevertheless, from an economic perspective, the agency is more independent due to the redesigning of the fee structure in 2017 with the self-funded part of the budget increasing from 70% in 2015 to 88% in 2018. Self-funding and independence are an important element for NRAs, and the independence was also stated in the TİTCK’s Strategic Plan 2019–2023 under the “Fundamental Principle and Value” section.

The general requirements of the marketing authorization applications in Turkey are in compliance with the ICH standards, and the CTD has been implemented as the content framework for submission dossiers since 2005. Regulations and technical guidelines to assist the industry were prepared in line with international standards including those of the EMA, US FDA, and WHO. As identified in the previous study, the TİTCK is only carrying out full reviews in the assessment of applications (Ceyhan et al., 2018). However, it is recommended that the agency should now consider the implementation of other regulatory review models such as the verification or abridged models, which are based on reliance and recognition of other reference agencies decisions, as this could reduce the approval timelines and support the TİTCK with its limited resources in managing the increasing workload (Liberti, 2017). Use of the reliance model is one of the criteria established by the WHO Benchmarking Study for agencies to become recognized reference agencies (World Health Organization, 2019). Therefore, the TİTCK should consider the possibility of implementing a reliance review model and update their legislation accordingly.

The structure of the TİTCK marketing authorization department was redesigned in 2016 and two major changes implemented. The first was the establishment of a separate unit responsible to conduct a final review check of the application following all the scientific assessments conducted by the expert committees. The second was related to the assignment of a new project management role to the internal TİTCK staff. Accordingly, every employee in the unit is responsible for a dedicated number of applications, this includes the internal monitoring of the application progress as well as managing all the questions and answers during the assessments. Furthermore, the structure of the scientific committees also changed in 2016, and internal experts are now chosen among agency staff according to their background, with individuals with post-graduate degrees such as Master of Science or Doctorates to be included in the committees. Thus, while the need and dependency on external experts has decreased, the organizational memory, knowledge management, expertise, and scientific evaluation capacity of the TİTCK have increased. According to the agency plan, the committees will consist of a majority of internal experts in the near future.

### TİTCK Review Timelines

This study identified the TİTCK regulatory review capacity at approximately 700 marketing authorization application per year. In 2018, 88% of all approvals were generics and the remainder were NASs. The approval numbers for generic applications increased from 561 in 2016 to 610 in 2018, whereas the NAS application numbers decreased from 2016 to 2018. This could be attributed to the local GMP accreditation process which can take several years if the application is not prioritized as well as the Turkish Pharmaceutical Pricing System, which employs a fixed exchange rate causing a 40% loss in the pricing of the medicines in comparison to the actual exchange rate. These two issues were perceived as major challenges for global companies, and therefore many companies may have been reluctant to submit NAS marketing authorization applications in Turkey. It is recommended that the TİTCK consider mutual recognition of other agencies’ GMP activities, especially with respect to PIC/s membership

**Figure 6:**
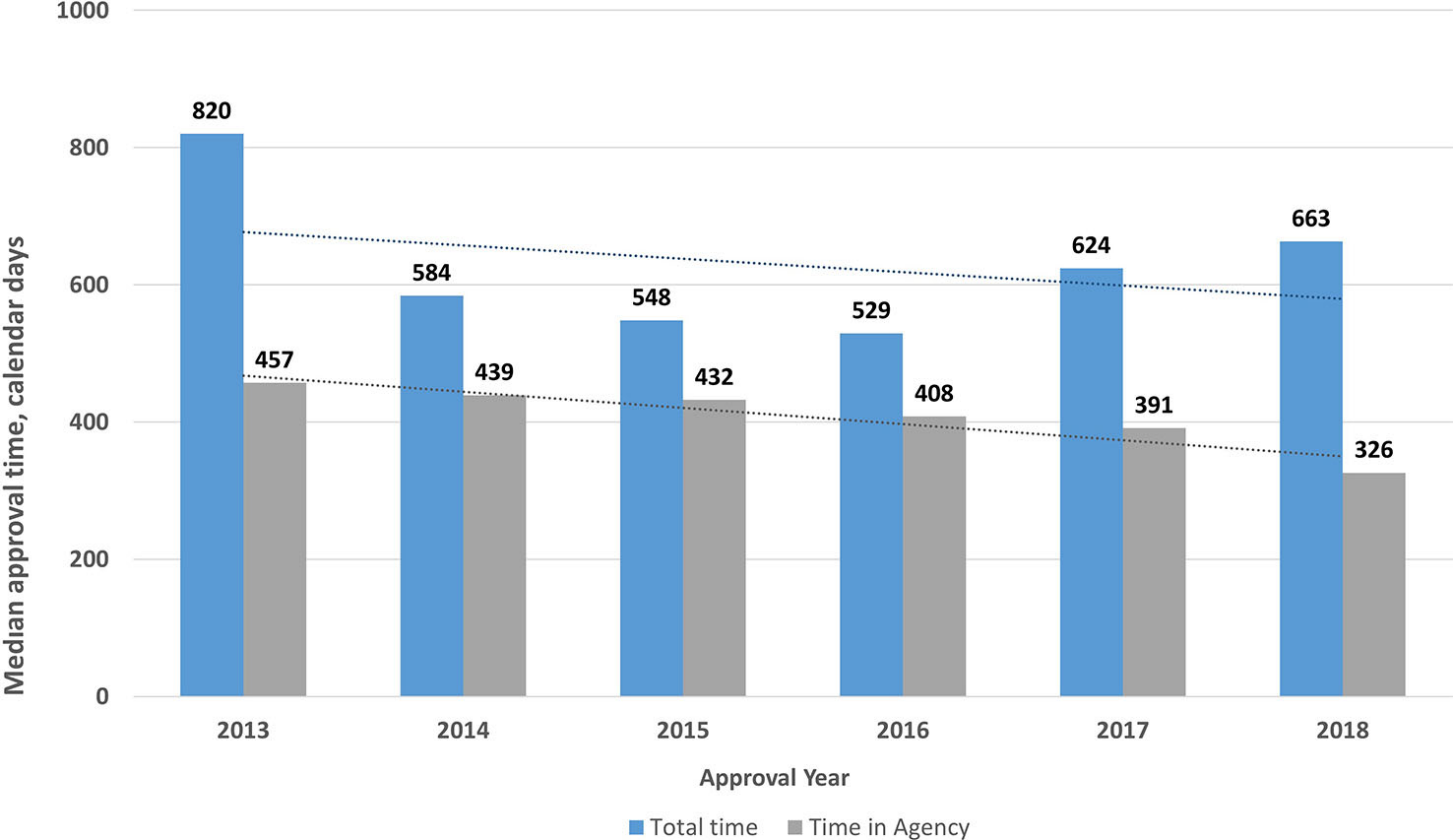
Median review timelines for new active substances (2013–2018).

The total review time for NASs from validation to final approval increased from 529 to 663 days in the period from 2016 to 2018, whereas between 2013 and 2015 the review time decreased from 820 to 548 days (**Figure 6**). Decreases in the total time as well as the time in the agency were statistically significant (P < .0001). This shows that there was no specific trend for the TİTCK approval timelines in the last six years. In terms of ATC class, similar to other agencies, antineoplastic and immunomodulating agents were 30% of the total the TİTCK NAS approvals from 2016 to 2018, which ranged from 553 days to 663 days (Bujar et al., 2018; Mullard, 2018). According to the TİTCK regulation, a marketing authorization application review should be completed within 210 days. This excludes clock stops for company response time, as there is no deadline for companies to respond according to the regulation.

However, it is of significance that the overall approval time within the agency between 2013 and 2018 decreased from 457 days in 2013 to 326 days in 2018 (**Figure 6**). Moreover, the queuing/backlog time decreased from 2 to 6 months to around 3 months for normal applications and from 2–8 weeks to 0 weeks for prioritized applications. These decreases came as a result of the management of the applications according to workload in the regulatory review process; however, despite these changes, the target approval timelines were still not achieved in the last 3 years, which indicates there is still room for improvement. It could be considered that providing pre-submission scientific advice for companies and setting deadlines for company response time could reduce the total time and enhance early patients’ access to medicines. The TİTCK has therefore included a provision in the draft marketing authorization regulation that requires company responses within a specified deadline and that enables pre-submission scientific advice. The review time within the agency is the key indicator of the TİTCK’s regulatory performance rather than the total approval time which includes the sponsor’s response time. However, the international standard of regulatory approval timelines for major agencies takes into consideration the time from submission to approval as the key indicator of patients’ access to innovative medicines (Bujar et al., 2018).

Prioritization has made a significant difference in review times. In 2016, the prioritization process was introduced by the TİTCK for the first time. Accordingly, target approval times for high-priority and priority applications were achieved in 2017, with 68 days for priority applications and 148 days for high-priority applications and in 2018 with 66 days for priority applications and 105 days for high-priority applications.

#### The TİTCK GRevP

The TİTCK has not formally implemented GRevP in all its review elements; however, some of these are informally covered by the agency, which has a plan to formally implement GRevP by 2021. One of the key elements of the GRevP is the use of a peer review, but the TİTCK still does not use this approach, which was a recommendation in the proposed new model from the previous study. However, a new similar practice has been adopted by the agency, which enables the expert committees’ decisions and related administrative documents to be reviewed by internal experts with the intent of a final check before granting of authorization. However, the TİTCK is still not utilizing structured assessment templates, which are considered important by other authorities such as the US FDA and EMA. Thus, the use of such an assessment template together with a structured approach for decision making would facilitate the preparation and publishing of public assessment reports. This topic was considered by the TİTCK within the proposed improved model from the previous study, and the agency is planning initially to publish a Turkey Public Assessment Report (TPAR) to be prepared by using assessment templates for some type of applications.

The TİTCK also plans to maintain consistency, increase internal scientific evaluation capacity and reduce approval time through the implementation of ATC-based evaluation by units specializing in specific prodcut groups. Furthermore, the agency plans to batch deficiencies identified during the assesment.

In terms of training, although the agency still does not have a formal framework, nevertheless informal induction training for external experts has been initiated since 2018. However, the completion of a training course is not required for professional advancement of internal and external experts.

While before 2016 transparency was assigned as “medium priority” by the agency, subsequently it has been assigned as “high priority”. This demonstrates that the agency has changed its approach to become more transparent in line with political will. Some examples initiated by the TİTCK to enhance transparency include periodic meetings with stakeholders, the initiation of an internal tracking system, and weekly department activity reports to manage application timelines and improvement of the external electronic tracking system to enable companies to follow the status of their applications. In addition, a regulation has been drafted that could facilitate the implementation of pre-application scientific advice meetings, which the TİTCK is planning to implement in the near future. Lastly, since the sharing of the summary basis of approval is a major issue in becoming more transparent, the TİTCK is also considering the possibility of implementing this in due course.

## Conclusions

To ensure patients’ timely access to medicines and to work more efficiently and effectively with all stakeholders, the TİTCK has made considerable efforts to improve its regulatory capacity since 2016 as a result of a number of recommendations. Some of the major changes initiated by the agency to improve the review system include redesigning the marketing authorization department, an investment in the agency’s human resources, and implementing elements of GRevPs. These developments have contributed to the overall decrease in the total review and approval times and enabled a better management of the workload/backlog in the agency, which was a major bottleneck in the past. While quantitively the agency has been successful, qualitatively there is still room for improvement if the TİTCK is to become an international reference agency in line with its current vision.

The TİTCK became a member of PIC/S in 2018, which was a major achievement, as membership in this international organization enables the agency to benefit from the GMP inspection outcomes and GMP certificates issued by other authorities, thus expediting the regulatory process through mutual recognition. In addition, as the TİTCK seeks to become an internationally recognized agency, it intends to move from observer status to a full membership in ICH as it seeks to comply with the implementation of international guidelines.

Implementation of other regulatory review models and pre-submission advisory meetings with companies, as well as setting a deadline for companies to respond to questions could reduce the total approval time of marketing authorization applications, which was relatively long in the past and affected patients’ access to medicines. The establishment of a project management system and peer review as well as initiating a formal training programme for agency staff and external experts including induction training and external courses could facilitate the formal implementation of GRevPs within the TİTCK. In terms of transparency, there are still areas of improvement with regard to pre-submission scientific advice meetings with companies as well as publishing a summary basis of approval. Finally, it is critical for the TİTCK to become a fully independent agency in order to reduce some of the barriers which would enable a faster and improved decision making process and to implement changes to improve the regulatory review process in a changing dynamic pharmaceutical environment.

### Recommendations

This study has identified key developments in the current TİTCK processes and practices highlighted in the recommendations from the previous paper which demonstrates the improved regulatory performance of the agency. Moreover, this study has also enabled several further suggestions to enhance patient’s access to medicines as well as to become an internationally recognized reference agency and a full ICH member. Therefore, the key recommendations from this study are to:

- Formalize the implementation of GRevPs within the agency and increase the awareness and knowledge of companies as well as external reviewers with regard to GRevP.
- Conduct laboratory analysis of new products after market authorization to reduce total approval time.
- Improve the transparency and consistency of the scientific review system by implementing a structured framework for decision making and benefit-risk assessment as well as publishing a summary basis of approval for stakeholders.
- Implement flexible regulatory review pathways, such as the verification and abridged reviews, by utilizing a reliance model in line with the WHO recommendation for Good Reliance Practices to order to conserve both resources and time.
- Enhance the legislative authority and independence of the TİTCK, which could minimize obstacles and expedite the issuance of new and updated regulatory guidelines/approaches in order to enable developments and necessary changes.
- Expedite the PIC/S mutual recognition process to improve the GMP accreditation process and facilitate access to innovative medicines.
- Establish a “Centre of Regulatory Excellence” in collaboration with international organizations, academia, and the pharmaceutical industry in order to conduct research in regulatory science and improve the local regulatory capacity and training frameworks of both the TİTCK staff and its external experts.

## Author Contributions

OK: Analyzed the data, wrote the manuscript. HG: Analyzed the data, wrote the manuscript. AA: Designed the study, wrote the manuscript. HC: Analyzed the data, wrote the manuscript. TP: Analyzed the data, wrote the manuscript. EM: Designed the study, analyzed data, wrote manuscript. SW: Designed the study, analyzed results, wrote manuscript.

## Conflict of Interest

The authors declare that the research was conducted in the absence of any commercial or financial relationships that could be construed as a potential conflict of interest.

## Abbreviations

CIRS, Centre for Innovation in Regulatory Science; CMC, chemistry, manufacturing, and controls; CPP, Certificate of Pharmaceutical Product; CTD, Common Technical Document; EMA, European Medicines Agency; FDA, Food and Drug Administration; GMP, good manufacturing processes; GrevP, good review practices; ICH, International Council for Harmonization of Technical Requirements for Pharmaceuticals for Human Use; MAA, marketing authorization application; NASs, new active substances; NRA, national regulatory authorities; PIC/S, Pharmaceutical Inspections and Cooperation Scheme; SOPs, standard operating procedures; TİTCK, Türkiye İlaç ve Tıbbi Cihaz Kurumu; TPAR, Turkish Public Assessment Report; WHO, World Health Organization.
