# Supplementary material for: Evaluation of the Performance of the Turkish Regulatory Agency: Recommendations for Improved Patients’ Access to Medicines
Source: Front Pharmacol. 2020 Feb 3;10:1557. doi: 10.3389/fphar.2019.01557 (PMC7008400; doi:10.3389/fphar.2019.01557)
Supplement: Supplementary file 1 [file DataSheet_1.pdf]

CONFIDENTIAL

ASSESSING THE REGULATORY REVIEW PROCESS IN EMERGING MARKETS

Key milestones, target times, and quality of decision making in the assessment and registration process

QUESTIONNAIRE

[Country]

August, 2019

CONTENTS

Background

Part 1 - Organisation of the agency

Part 2 - Types of Review Models

Part 3 - Key Milestones in the Review Process

Part 4 - Good Review Practices (GRevP): Building quality into the assessment and registration process

Part 5 – Quality Decision-Making Processes

Glossary and abbreviations

The Centre for Innovation in Regulatory Science (CIRS)

CIRS - The Centre for Innovation in Regulatory Science Limited - is a neutral, independently managed UK-based subsidiary company, forming part of Clarivate Analytics (UK) Limited. CIRS' mission is to maintain a leadership role in identifying and applying scientific principles for the purpose of advancing regulatory and HTA policies and processes. CIRS provides an international forum for industry, regulators, HTA and other healthcare stakeholders to meet, debate and develop regulatory and reimbursement policy through the innovative application of regulatory science and to facilitate access to medical products through these activities. This is CIRS' purpose. CIRS is operated solely for the promotion of its purpose. The organisation has its own dedicated management and advisory boards, and its funding is derived from membership dues, related activities, special projects and grants.

Centre for Innovation in Regulatory Science (CIRS)
Friars House, 160 Blackfriars Road, London SE1 8EZ, United Kingdom

Email: cirs@cirsci.org

Website: www.cirsci.org

Confidentiality

CIRS recognises that much of these data may be highly sensitive. CIRS has more than 20 years of experience in handling similar data provided by agencies regarding individual products in regulatory review. **All information collected from individual agencies will be kept strictly confidential. No data that will identify an individual agency will be reported or made available to any third party.** External reports or presentations of the data will include only blinded results and any appropriate analytical interpretations.

ASSESSING THE REGULATORY REVIEW PROCESS IN EMERGING MARKETS

Review of key milestones, target times and quality of decision-making in the assessment and registration process

BACKGROUND

This questionnaire supports an on-going programme by CIRS, focusing on the regulation of new medicines in emerging markets, and looking at how regulatory agencies build quality into their review process.

The first phase was initiated in January 2004 to assess the regulatory environment in some 30 countries, using comparative data, at the country and regional level, to identify the key issues for improving review practices and making new medicines available in an efficient and timely manner. Some of these, for example, the timing and use of the Certificate of a Pharmaceutical Product (CPP) and the length of the review process, were analysed in detail. This project highlighted the need to understand more about the different steps in the review process and the way in which these affect the overall timeline. Regulatory authorities also showed an interest in having a greater understanding of how agencies are building quality into the review process.

Through this on-going programme, CIRS maps the key milestones and associated activities, for each participating agency, for new marketing applications, and to identify the processes and procedures associated with the implementation of Good Review Practices (GRevP) that help build quality into the review process. This provides a platform to enable information sharing across agencies.

This questionnaire has been designed to collate information in a single place; agencies may have collected some of these data for other assessment (benchmarking) projects. However, this project has several unique aspects:

- It collects all the key information in a single document from which a consolidated Country Report will be created;
- It allows the metrics that are collected here and in the future to be related to the PROCESS that the agency uses thereby allowing for a more qualified assessment;
- It is part of a global programme called Optimising Efficiencies in Regulatory Agencies (OpERA), coordinated by CIRS on behalf of regulatory agencies around the world. The milestones and questions have been carefully crafted to be relevant to any agency - large or small, mature or maturing - to provide relevant data that can be used for internal purposes or as applicable, for agency-to-agency comparisons. For example, see Emel Mashaki Ceyhan et al: The Turkish Medicines and Medical Devices Agency: Comparison of Its Registration Process with Australia, Canada, Saudi Arabia, and Singapore. *Frontier's in Pharmacology* January 2018, Volume 9, Article 9.

OBJECTIVES

The objectives of this on-going programme are to:

- Identify the key milestones and target times for each agency and the main activities between milestones;
- Identify the model(s) of the review which is being undertaken by each agency;
- Identify opportunities for the exchange of better practices amongst regulatory authorities;
- Assess how agencies are building quality into the assessment and registration processes.

OUTPUT

Participating agencies will receive a Country Report derived from the data provided in this Questionnaire, with which they can compare their regulatory procedures with those of peer agencies across regions. This includes an analysis of where time is spent in the review process.

The outcome allows an analysis of the quality measures that are in place for a certain type of review, and provides a baseline for subsequent comparative studies across agencies to establish best practices.

ABOUT THE QUESTIONNAIRE

The attached questionnaire is divided into four sections:

Part 1: Organisation of the agency. The **Introduction** to the questionnaire asks the agency to provide current information on its structure, organisation and resources.

Part 2: Types of review models: Explores ***review model(s)*** for the ***scientific assessment of medicines*** in terms of the extent to which data is assessed in detail by the agency, and how the agency might rely on the results of assessments and reviews carried out elsewhere.

Part 3: Key milestones in the review process. This part of the questionnaire is based on the General Model, giving a process map and milestones, that has been developed from studying procedures followed in 'established' and 'emerging' regulatory agencies. It captures the main steps in the review and approval process and identifies key 'milestone' dates in the process. This allows for the analysis of timelines.

Part 4: Good Review Practices (GRevP): Building quality into the regulatory process looks at the activities that contribute to those measures that have been adopted to improve consistency, transparency, timeliness, and competency in the review processes.

Part 5: Quality Decision-Making Processes. This part of the questionnaire explores to the quality of the decision-making process and whether or not the agency has measures in place to ensure that good decisions are made around the data during the registration process.

Where appropriate, additional information may be obtained during face-to-face agency-CIRS interactions.

Focus of the Questionnaire

This questionnaire is intended, primarily, to document procedures and practices that relate to medicines that are the subject of **major** applications; i.e., new active substances and major line extensions.

New Active Substance

A new chemical, biological, or pharmaceutical active substance including:

- a chemical, biological, or radiopharmaceutical substance not previously authorised as a medicinal product;
- an isomer, mixture of isomers, a complex or derivative or salt of a chemical substance not previously authorised as a medicinal product, but differing in properties regarding safety and efficacy from that chemical substance previously authorised;
- a biological substance previously authorised as a medicinal product, but differing in molecular structure, nature of the source material or manufacturing process;
- a radiopharmaceutical substance which is radio nucleotide, or a ligand not previously authorised as a medicinal product, or the coupling mechanism to link the molecule and the radio nucleotide has not been previously authorised.

Major line extension

A major line extension is a change to an authorised Medicinal Product that is sufficiently great that it cannot be considered as a simple variation to the original product, but requires a new product authorisation. Such changes include major new therapeutic indications or new disease states, extension to new patient populations (e.g., paediatrics), a new route of administration or a novel drug delivery system.

PART 1. ORGANISATION OF THE AGENCY

As background to the discussions about your agency, its practices and procedures it would be helpful to have the following basic information on its structure and the way it is organized:

***Title of the Agency/Division** responsible for the regulation of medicinal products for human use:*

If this is part of a parent agency with a wider remit (e.g., food and drugs) please give the title:

About the agency

1.1 Indicate which of the following best describes this agency:

☐ Autonomous agency, independent from the Health Ministry administration
☐ Operates within the administrative structure of the Health Ministry

Date of establishment of the current agency: ☐

Scope of Activities

1.2 Please indicate the scope of responsibility of the agency:

Medicinal products for human use	☐ **YES**	☐ **NO**
Medicinal products for veterinary use	☐ **YES**	☐ **NO**
Medical devices and *in vitro* diagnostics	☐ **YES**	☐ **NO**

1.3 Indicate the main activities that are covered by the agency:

☐ Marketing authorisations/product licences
☐ Post-marketing surveillance
☐ Laboratory analysis of samples
☐ Other: Site inspections (site visits)
☐ Clinical trial authorisations
☐ Regulation of advertising
☐ Price regulation

Budget / Funding

Please indicate whether the following data:

☐ **are in the public domain**

☐ **should be treated as confidential**

1.4 Please provide the following information on the agency budget for the regulation of medicinal products for human use:

	**Local currency (please specify)**	**US$**
☐ **Total annual budget**		
☐ **Year for which data are given**		

☐ **If the budget is sub-divided according to different activities, please specify:**

% of total budget

1.4 Please provide the following information on the agency budget for the regulation of medicinal products for human use:

☐ **Clinical trial authorisations**	
☐ **Marketing authorisations**	
☐ **Pharmacovigilance**	
☐ **Other post-marketing controls**	
☐ **Other activities (Please specify):**	

Sources of funding

1.5 Please provide the following information in relation to the way the agency is funded:

Funded entirely by the government	☐ **YES**	☐ **NO**
Self-funded entirely from fees	☐ **YES**	☐ **NO**
Partially funded from different sources (please give proportions of total budget)	**% Government**<br>**% Other (please specify)**	**% Fees**

Review team

Please note that the following questions refer to the regulation of **medicinal products for human use.**

1.6 Please provide information on staff numbers:

• Total staff in the agency	
• Total number of reviewers for applications for marketing authorisations/ product licences	
• Number of reviewers for applications for marketing authorisations/ product licences or synthetic and biological products	

1.7 Please indicate the professional background and numbers of the ***technical*** *agency staff assigned to the review and assessment of medicinal products:*

	Number employed as assessors (degree/expertise)			
	Total	with PhD or PharmD	with Masters Degree	Other
• Physicians				
• Statisticians				
• Pharmacists				
• Other Scientists				
• Project Managers				

Fees charged for review applications

1.6 Are fees charged to sponsors for the review and assessment of applications for medicinal products for human use? ☐ **YES** ☐ **NO**

If **YES**, please provide the following information:

Marketing Authorisation Application fee for:	**Local currency (please specify)**	**US$ (rounded)**
☐ New Active Substance synthesis		
☐ New Active Substance biological		
☐ Established ingredient - proprietary product synthesis		
☐ Established ingredient - proprietary product biological		
☐ Generic product		
☐ Biological competitor product		
☐ Variations		
☐ Major line extension		
☐ Other (Please specify)		
Does the agency charge a fee for scientific advice? ☐ **YES** ☐ **NO** If **YES,** please provide		

1.7 Applications received

**Type**	**Number of applications received in each year**			**Current backlog**
	**2016**	**2017**	**2018**	
New Active Substance				
Major line extension				
Generics (all)				
WHO Pre-qualified generics (if applicable)				

1.8 Applications determined

**Type**	**Number of applications determined in each year**		
	**2016**	**2017**	**2018**
New Active Substances approved			
New Active Substances refused			
Major line extensions approved			
Major line extension refused			
Generics approved			
Generics refused			
WHO Pre-qualified generics approved			
WHO Pre-qualified generics refused			

Additional documentation

*To assist CIRS to better understand your organisation, please provide copies of any **organisation charts** that show the structure of the agency and its relationship to other regulatory bodies; e.g., medical device agency. It would also be very useful to have copies of any background papers that describe the **functions**, **remit,** and **mission** of the agency.*

PART 2. TYPES OF REVIEW MODELS

Three basic types of scientific review have been identified. Many agencies apply a different level of data assessment to different applications, according to the type of product and/or its regulatory status with other agencies. The data assessment models for scientific review are described in section 2.1 below and further questions are set out in 2.2 to analyse the types of scientific review in more detail.

2.1 Please indicate by checking the boxes below, which descriptions fit the model(s) used by your agency in the assessment of major applications i.e., new active substances (NASs) and major line extensions as described earlier.

Data Assessment Type 1 (Verification)

This model is used to reduce duplication of effort by agreeing that the importing country will allow certain products to be marketed locally once they have been authorised by one or more recognised reference agencies, elsewhere. The main responsibility of the agency in the importing country is to 'verify' that the product intended for local sale has been duly registered as declared in the application and that the product characteristics (formulation, composition) and the prescribing information (use, dosage, precautions) for local marketing conforms to that agreed in the reference authorisation(s).

**TYPE 1**	☐ Not used<br>☐ Used for selected applications (please specify):	☐ Used for all major applications
**Comment:**		

Data requirements for Type 1 Assessments (verification)- What do you review/assess?

CPP/Public assessment reports/un-redacted assessment reports/Free sales certificate/etc	
Similarity to registered product	
Quality data	
Non-clinical data	
Clinical data	
Local benefit-risk assessment	

Data Assessment Type 2 (Abridged)

This model also conserves resources by not re-assessing scientific supporting data that has been reviewed and accepted elsewhere but includes an 'abridged' independent review of the product in terms of its use under local conditions. This might include a review of the pharmaceutical (CMC) data in relation to climatic conditions and distribution infrastructure and a benefit-risk assessment in relation to use in the local ethnic population, medical practice/culture and patterns of disease and nutrition.

Approval by a recognised agency elsewhere is a pre-requisite before the local authorisation can be granted but the initial application need not necessarily be delayed until formal documentation such as a Certificate of a Pharmaceutical Product (CPP) is available.

**TYPE 2** **Comment:**	☐ Not used ☐ Used for all major applications

Data requirements for Type 2 Assessments (abridged) **What do you review/assess?**

**CPP/Public assessment reports/un-redacted assessment reports/Free sales certificate/etc**	
**Similarity to registered product**	
**Quality data**	
**Non-clinical data**	
**Clinical data**	
**Local benefit-risk assessment**	

Data Assessment Type 3 (Full)

In this model the agency has suitable resources, including access to appropriate internal and external experts, to carry out a 'full' review and evaluation of the supporting scientific data (quality, pre-clinical, clinical) for a major application. A Type 3 assessment could be carried out on a new application that has not been approved elsewhere but, in practice, legal requirements may dictate that the product must be authorised by a reference agency before the local authorisation can be finalised.

**TYPE 3**	☐ Not used ☐ Used for all major applications<br>☐ Used under the following conditions (please specify):<br>☐ Full review conducted but product must still be authorised by a reference agency prior to final authorisation

Data requirements for Type 3 Assessments (full) What do you review/assess?

**CPP/Public assessment reports/un-redacted assessment reports/Free sales certificate/etc**	
**Similarity to registered product**	
**Quality data**	
**Non-clinical data**	
**Clinical data**	
**Local benefit-risk assessment**	

If your agency has **recognised 'reference agencies'** (as may be used for reliance or recognition in Types 1 and 2 reviews) please list the countries/agencies/authorities:

PRIORITY/FAST TRACK PRODUCTS

Does your company have available:

☐ A priority review track

☐ A fast track (if different from priority)

2.2 Data requirements and assessment

	Type 1	Type 2	Type 3	Priority/fast track products
Evidence of authorisation by other authorities				
Requirements for a CPP as part of the review	☐ with application<br>☐ before authorisation<br>☐ not essential	☐ with application<br>☐ before authorisation<br>☐ not essential	☐ With the application and before local authorisation<br>☐ not essential<br>☐ If available at the time of submission	☐ with application<br>☐ before authorisation<br>☐ not essential
Other documentation from the authorising agencies accepted as evidence of registration	☐ letter of authorisation<br>☐ copy of full authorisation<br>☐ Internet evidence	☐ letter of authorisation<br>☐ copy of full authorisation<br>☐ Internet evidence	☐ letter of authorisation<br>☐ copy of full authorisation<br>☐ Internet evidence<br>☐ None	☐ letter of authorisation<br>☐ copy of full authorisation<br>☐ Internet evidence<br>☐ None
Other evidence accepted				
Verification of identity between the authorised product and the local application				
	**Type 1**	**Type 2**	**Type 3**	
	**Information must be:**<br>***Identical*** ***Closely similar***	**Information must be:**<br>***Identical*** ***Closely similar***	**Not applicable**	
Dosage form	☐ ☐	☐ ☐		
Strength	☐ ☐	☐ ☐		

	Type 1	Type 2	Type 3	Priority/fast track products
Ingredients	☐ ☐	☐ ☐		
Indications and dosage	☐ ☐	☐ ☐		
Warnings and precaution	☐ ☐	☐ ☐		
Product label	☐ ☐	☐ ☐		
Product name	☐ ☐	☐ ☐		
Other (specify)				
Scientific data required to support the application (Reference is made below to sections of the ICH Common Technical Document (CTD) as an example of the level of detail but does not imply that the CTD in necessarily accepted).				
Pharmaceutical quality/CMC	☐ Summary data (Mod 2.3)<br>☐ Summary + full stability<br>☐ Full data (Mod 3)	☐ Summary data (Mod 2.3)<br>☐ Summary + full stability<br>☐ Full data (Mod 3)	☐ Summary data (Mod 2.3)<br>☐ Summary + full stability<br>☐ Full data (Mod 3)	☐ Summary data (Mod 2.3)<br>☐ Summary + full stability<br>☐ Full data (Mod 3)
Scientific data required to support the application (continued)				
Non-clinical data	☐ Written summary (2.4)<br>☐ Tabulated data (2.5)<br>☐ Full data (Module 4)	☐ Written summary (2.4)<br>☐ Tabulated data (2.5)<br>☐ Full data (Module 4)	☐ Written summary (2.4)<br>☐ Tabulated data (2.5)<br>☐ Full data (Module 4)	☐ Written summary (2.4)<br>☐ Tabulated data (2.5)<br>☐ Full data (Module 4)
Clinical data	☐ Written summary (2.5)<br>☐ Tabulated data (2.6)<br>☐ Full data (Module 5)	☐ Written summary (2.5)<br>☐ Tabulated data (2.6)<br>☐ Full data (Module 5)	☐ Written summary (2.5)<br>☐ Tabulated data (2.6)<br>☐ Full data (Module 5)	☐ Written summary (2.5)<br>☐ Tabulated data (2.6)<br>☐ Full data (Module 5)
Extent of Scientific Review				
Quality/CMC data	☐ Only examined if there is a query<br>☐ 'Check list' review for completeness of data<br>☐ Selective review in detail (e.g. stability, specification)<br>☐ Detailed assessment and evaluation report	☐ Only examined if there is a query<br>☐ 'Check list' review for completeness of data<br>☐ Selective review in detail (e.g. stability, specification)<br>☐ Detailed assessment and evaluation report	☐ 'Check list' review for completeness of data<br>☐ Selective review in detail (e.g. stability, specification)<br>☐ Detailed assessment and evaluation report	☐ Only examined if there is a query<br>☐ 'Check list' review for completeness of data<br>☐ Selective review in detail (e.g. stability, specification)<br>☐ Detailed assessment and evaluation report

	Type 1	Type 2	Type 3	Priority/fast track products
**Comments:**				
Non-clinical data	☐ Only examined if there is a query<br>☐ 'Check list' review for completeness of data<br>☐ Detailed assessment and evaluation report	☐ Only examined if there is a query<br>☐ 'Check list' review for completeness of data<br>☐ Detailed assessment and evaluation report	☐ 'Check list' review for completeness of data<br>☐ Detailed assessment and evaluation report<br>☐ Not at all	☐ Only examined if there is a query<br>☐ 'Check list' review for completeness of data<br>☐ Detailed assessment and evaluation report
**Comments:**				
Clinical data	☐ Only examined if there is a query<br>☐ 'Check list' review for completeness of data<br>☐ Selective review in detail (e.g. bridging studies)<br>☐ Detailed assessment and evaluation report	☐ Only examined if there is a query<br>☐ 'Check list' review for completeness of data<br>☐ Selective review in detail (e.g. bridging studies)<br>☐ Detailed assessment and evaluation report	☐ 'Check list' review for completeness of data<br>☐ Selective review in detail (e.g. bridging studies)<br>☐ Detailed assessment and evaluation report	☐ Only examined if there is a query<br>☐ 'Check list' review for completeness of data<br>☐ Selective review in detail (e.g. bridging studies)<br>☐ Detailed assessment and evaluation report
**Comments:**				

Clinical evaluation: factors included in the risk-benefit assessment

The clinical opinion takes account of:	**Never**	**Sometimes**	**Always**	**Never**	**Sometimes**	**Always**	**Never**	**Sometimes**	**Always**	**Never**	**Sometimes**	**Always**
Differences in medical culture/practice	☐	☐	☐	☐	☐	☐	☐	☐	☐	☐	☐	☐
Ethnic factors	☐	☐	☐	☐	☐	☐	☐	☐	☐	☐	☐	☐
National disease patterns	☐	☐	☐	☐	☐	☐	☐	☐	☐	☐	☐	☐
Unmet medical need	☐	☐	☐	☐	☐	☐	☐	☐	☐	☐	☐	☐

	**Type 1**			**Type 2**			**Type 3**			**Priority/fast track products**		
Additional information, not in the application:												
The agency tries to obtain:	**Never**	**Sometimes**	**Always**	**Never**	**Sometimes**	**Always**	**Never**	**Sometimes**	**Always**	**Never**	**Sometimes**	**Always**
Other agencies' internal assessment reports	☐	☐	☐	☐	☐	☐	☐	☐	☐	☐	☐	☐
Reports available on the Internet (e.g., EPARS)	☐	☐	☐	☐	☐	☐	☐	☐	☐	☐	☐	☐
General Internet search	☐	☐	☐	☐	☐	☐	☐	☐	☐	☐	☐	☐
Other data (please specify):	☐	☐	☐	☐	☐	☐	☐	☐	☐	☐	☐	☐

PART 3. KEY MILESTONES IN THE REVIEW PROCESS

Review Process Map and Milestones

This part of the questionnaire is based on the General Model below, giving a process map and milestones that have been developed from studying procedures followed in 'established' and 'emerging' regulatory agencies. It captures the main steps in the review and approval process and identifies key 'milestone' dates in the process for monitoring and analysing timelines.

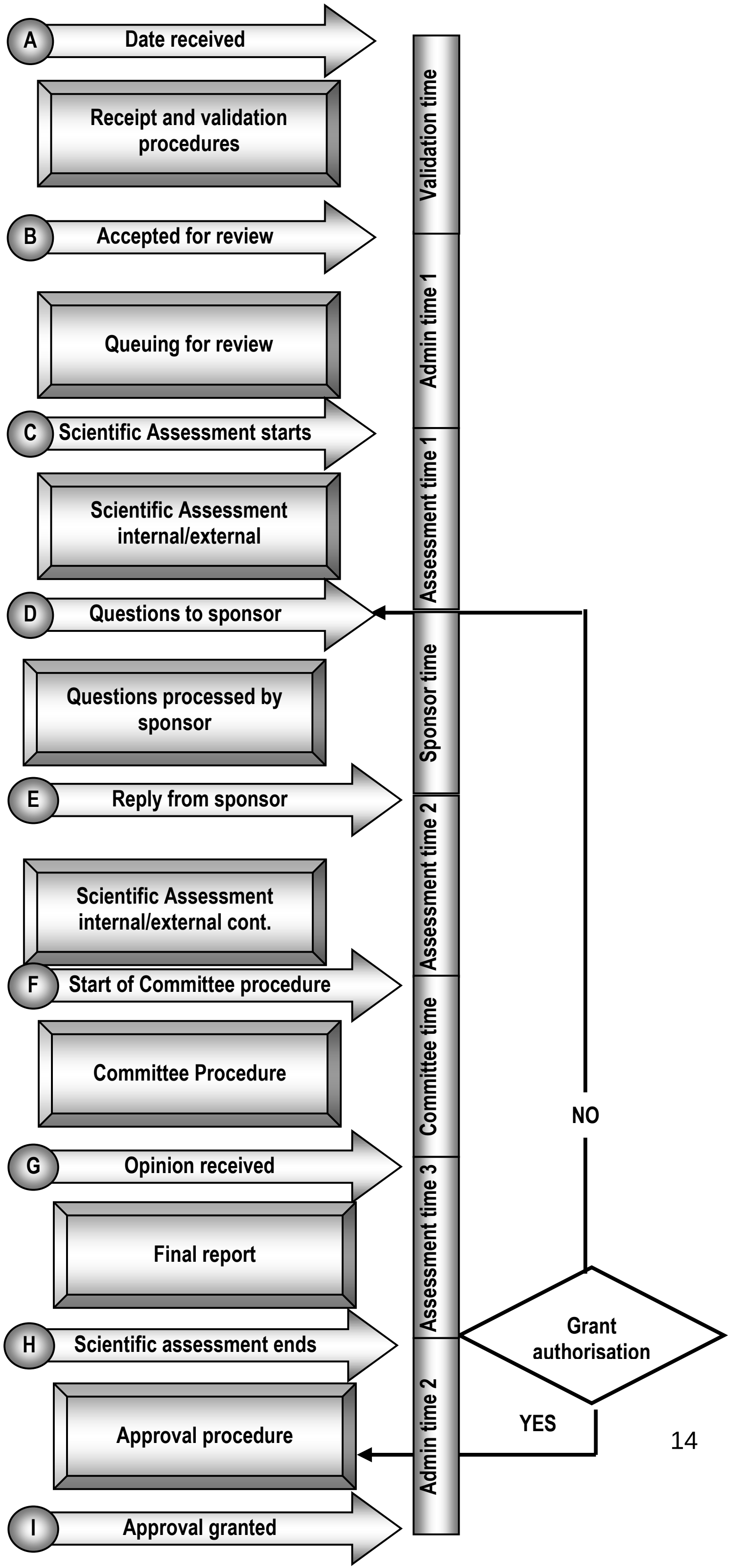

Notes

Receipt and validation may include administrative registration (reference number) and checks on legal requirements, status of company, local agent, manufacturer etc. as well as a 'checklist' validation of the application content (e.g., technical sections, CPP status).

Queuing for review: *Administrative time 1* is a measure of the 'backlog' time (if any) while valid applications wait for action to begin.

Scientific Assessment extends from milestone C to milestone H and is a measure of 'review time.' In some systems, the 'clock' stops when questions are asked and Sponsor ***time*** (milestone D to milestone E) can be measured and deducted from the agency review time.

Questions to sponsor may be batched and sent at one time or asked throughout the review process, in which case the *Sponsor time* is not easily measured.

In some systems, questions may only be sent to the sponsor after the end of the 'first cycle' scientific assessment (at milestone H).

Committee Procedure: Most review procedures for major applications include a step where the opinion of an expert advisory committee is sought. In this scheme, the Committee procedure is 'nested' within the Scientific Assessment but it may take place after the Agency's scientific assessment is complete.

Second cycle: If the application cannot be granted immediately, on technical grounds, it enters a second review cycle (new data point D: questions to sponsor) and a further scientific assessment is made of the additional data. The Committee Procedure may or may not need to be included in the second and subsequent review cycles.

Approval procedure: The time interval after scientific review (*Admin time 2*) while the formal authorisation is issued may be extended by pricing negotiations and finalisation of analytical and GMP checks.

Approval time is measured from milestone A to milestone I.

Review stages and milestones

This section of the questionnaire is based on the ***General Model*** shown on page 14.

> We recognise that not all systems conform to the general model and it would be very helpful if you could provide an outline of the model used by your agency. If this differs according to the **Type of data assessment** (see page 8) please provide information on the different models.

When information is given on target or actual times please indicate here whether these are counted in:

☐ **Calendar days** ☐ **Working days**

When 'milestone' dates are recorded during the review process is the information entered into an electronic tracking/recording system?

☐ **YES,** a system is in current use ☐ **NO,** a system is in development (please specify target date):

☐ **NO,** a manual system will be used for the foreseeable future

3.1 Receipt and Validation

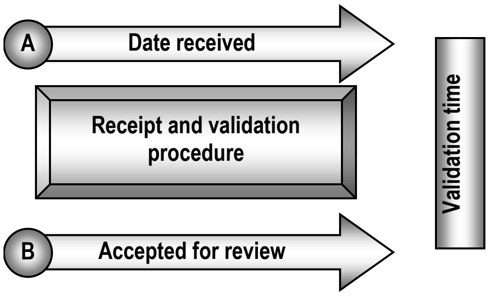

Pre-submission requirements

3.1.1 Are there any formal requirements before an application is submitted, for example, notification of intent to submit, assignment of registration code etc.,?

☐ **NO** ☐ **YES** (please specify):

Validation

3.1.2 Is the date of receipt (milestone A) formally recorded?	☐ **YES**	☐ **NO**
3.1.3 Are the following administrative items checked in the pre-review validation process?		
• Legal status of applicant/local agent	☐ **YES**	☐ **NO**
• GMP status of manufacturer	☐ **YES**	☐ **NO**
• Patent/IP status of active ingredient	☐ **YES**	☐ **NO**
• Whether company has paid the correct fee	☐ **YES**	☐ **NO**
**Other:**		

3.1.4 For those applications ***where prior authorisation elsewhere is essential*** *(**see Section 2**) please answer the following questions about the* ***Certificate of a Pharmaceutical Product*** (CPP):

Is the inclusion of a CPP an absolute requirement before accepting the application as valid?

☐ **YES** ☐ **NO** ☐ For some applications (please specify):

If **YES,** must the CPP be legalised by an Embassy or Consulate?	☐ **YES**	☐ **NO**
If **NO**, please indicate which of the following apply:		
• A CPP must be provided before the authorisation is issued	☐ **YES**	☐ **NO**
• Other evidence of authorisation by other countries is accepted in place of the CPP (e.g., copy of authorisation, Internet reference)	☐ **YES**	☐ **NO**

Comments:

Validation *(cont.)*

3.1.5 Is the application also checked for the following items?

Acceptable format (e.g. ICH CTD or local requirements)	☐ **YES** ☐ **NO**
Correct sections of scientific data (quality, safety, efficacy)	☐ **YES** ☐ **NO**
Other technical items:	

Acceptance for review/refusal to file

3.1.6 Is the date of acceptance (milestone B) formally recorded? ☐ **YES** ☐ **NO**

3.1.7 What happens if the application is incomplete?

☐ **Refusal to file:** New application must be made

☐ **File pending:** A request for the missing data is sent to the applicant
What is the time limit for the applicant to reply?

Comments:

Target time for validation

3.1.8 Is there a target validation time? ☐ **YES** ☐ **NO**

If **YES**, please specify:

3.2 Queuing/backlog

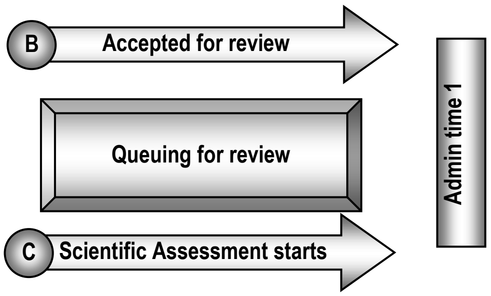

3.2.1 Which of the following applies to the queuing system for new applications?

☐ Held in queue after validation (as in the General Model) after phase 1 validation

☐ Held in queue before validation starts (milestone A)

3.2.2 What is the current queue time (approximately)?

☐ Less than 2 weeks ☐ 2-8 weeks

☐ 2-6 months ☐ 6 months-1 year

☐ More than 1 year

3.2.3 Are priority products taken out of turn in the queuing system?

☐ **YES,** always

☐ **YES,** sometimes

☐ **NO,** all applications await their turn

Comments:

3.2.4 Does the agency regard the backlog of applications as a problem? ☐ **YES** ☐ **NO**

If **YES**, how is this being addressed:

3.3 Scientific Assessment

3.3.1 Initiation of scientific review

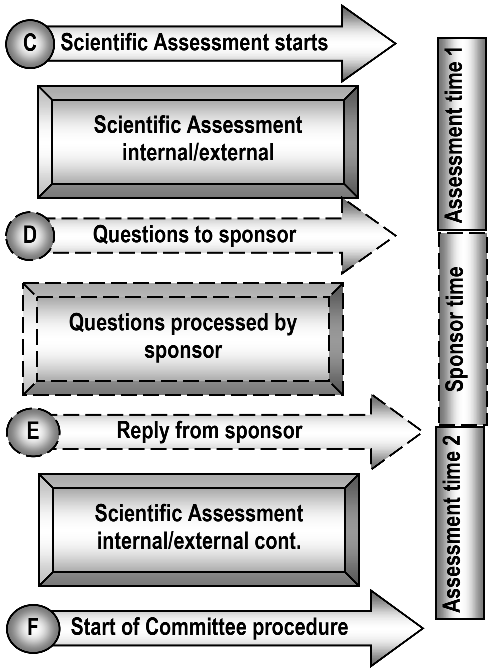

3.3.2 Is the start of the Scientific Assessment formally recorded (milestone C)? ☐ **YES** ☐ **NO**

3.3.3 Is the scientific data separated into three sections (quality, safety, and efficacy) for review? ☐ **YES** ☐ **NO**

3.3.4 In what order are the different sections assessed?

☐ In parallel ☐ In sequence

If in sequence, please give order:

*3.3.5 Who carries out the **primary** scientific assessment?*

☐ Agency technical staff ☐ Sent to outside experts

☐ Different procedure for different sections

Please describe the process:

3.4 Use of outside experts

If outside experts are used for the assessment of scientific data (C above) please complete the following:

3.4.1 Number of experts on the agency's list or panel: ☐

3.4.2 Main responsibility:

- ☐ To provide a detailed assessment report and recommendation
- ☐ To provide a clinical opinion on the product
- ☐ To provide advice to the agency staff on specific technical issues
- ☐ Other (Please specify):

3.4.3 Is there a contractual agreement on working within deadlines set by the agency? ☐ **YES** ☐ **NO**

3.5 Interactions with the Sponsor

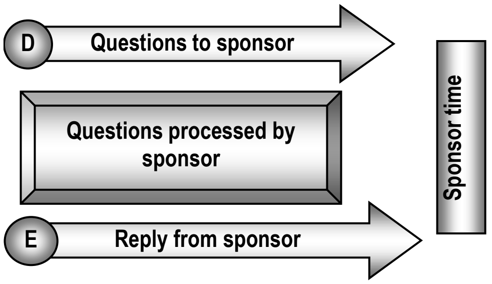

3.5.1 How are questions sent to the Sponsor?

☐ As they arise during the assessment ☐ Collected into a single batch

3.5.2 When are batched questions sent to the Sponsor?

- ☐ After the initial assessment but before reporting to the Scientific Committee (as in the General model)
- ☐ Not until the Scientific Committee has given its advice
- ☐ Before and after reference to the Scientific Committee

3.5.3 Does the scientific review cease while questions are being processed by the Sponsor ('clock stop')?	☐ **YES** ☐ **NO**

3.5.4 Can the sponsor time be calculated, i.e., are milestones D and E recorded?	☐ **YES** ☐ **NO**
3.5.5 Is the sponsor given a time limit to reply?	☐ **YES** ☐ **NO**
If **Yes**, what time is allowed?	

Meetings

3.5.6 Can the Sponsor hold meetings with the agency staff to discuss questions and queries that arise during the assessment?	☐**YES** ☐ **NO**
If **Yes**, what conditions and restrictions (if any) are applied:	

3.6 Review by Scientific Committee(s)

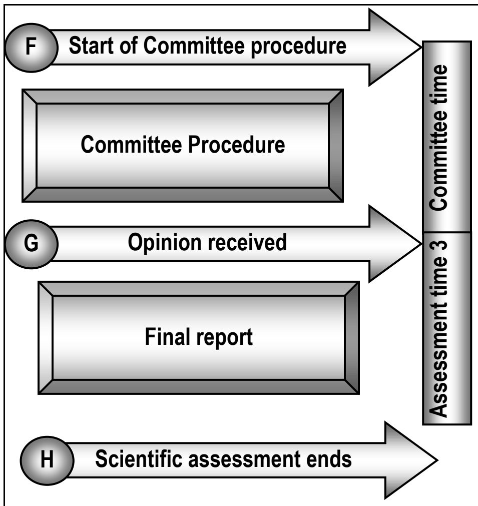

3.6.1 Is a Committee of Experts (internal and/or external) used in the review process? ☐ **YES** ☐ **NO**

*3.6.2 If **YES**, at which stage in the review?*

- ☐ Responsible for the whole assessment of the dossier from the start of the review
- ☐ Integrated into the agency's own internal/external scientific review procedure
- ☐ Consulted after the agency has reviewed and reported on the scientific data
- ☐ Other (Please specify):

3.6.3 Are the dates at the start and end of the Committee Review recorded (milestones F and G)?	☐ **YES** ☐ **NO**
3.6.4 Is the agency mandated to follow the Committee recommendation?	☐ **YES** ☐ **NO**
3.6.5 Is there a time limit for the Committee Procedure?	☐ **YES** ☐ **NO**
If **YES**, please give the target:	
If **NO**, what is the time range?	
3.6.6 Is there an additional step in the scientific review process, after the Committee has given its opinion?	☐ **YES** ☐ **NO**
If **YES**, please describe briefly the work carried out at this stage (e.g., final report and agency opinion).	

If **NO**, the milestone G will mark the end of the scientific review for the purpose of calculating the review time.

Target timelines for the review process

3.6.7 Is a target time set for the scientific review (milestones C to H)?		☐ **YES** ☐ **NO**
If **YES** please give target		

3.7 Recommendation on the Application

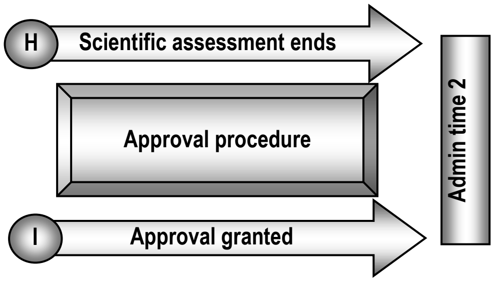

At the end of the Scientific Review (see General Model, page 19) there is normally recommendation that either:

- The product meets the scientific criteria for authorisation (proceed to approval procedure) *or*
- Further data is required before the scientific criteria are met (application enters a **second cycle** at milestone D (questions to Sponsor) *or*
- The application should be refused (not shown in the General Model)

3.7.1 Responsibility for the authorisation decision

3.7.1.1 Who makes the decision that a marketing authorisation can be granted?

☐ The Scientific Committee ☐ The Head of the Agency

☐ The Minister of Health

☐ Other (please specify):

3.7.1.2 If Scientific Advice Committee is used as per 3.7.1.1, what kind of decision-making process is used?

☐ Consensus process by the Committee

☐ Majority vote by the Committee

☐ One individual makes the final decision based on the Committee recommendations

☐ Other, please specify…………

3.7.2 Other criteria to be met

*3.7.2.1 Is the issue of the authorisation dependent on a **pricing agreement?*** ☐ **YES** ☐ **NO**

If **YES**, when are the pricing negotiations started?

☐ At the start of the scientific review ☐ After the end of the scientific review

☐ After the start but before the end of the scientific review

*3.7.2.2 Is the issue of the authorisation dependent on **sample analysis?*** ☐ **YES** ☐ **NO**

If **YES**, when is the analytical work started?

☐ In parallel with the scientific review ☐ At the end of the scientific review

☐ After the start, but before the end of the scientific review

*3.7.2.3 Is there a separate negotiation of the **product labelling/ product information** after the scientific opinion is given but before the approval is issued?*<br>**Comments:**	☐ **YES** ☐ **NO**
3.7.2.4 Please specify any other legal/administrative matters that must be finalised before the approval can be issued:	

3.7.2.5 Is the sponsor informed of a positive scientific opinion at milestone G, i.e., before the authorisation is issued?	☐ **YES** ☐ **NO**
3.7.2.6 Approximately how long does it take from receiving a positive scientific opinion (at milestone H) to issuing an approval (milestone I)?<br>☐ Less than a month ☐ 1-3 months ☐ 3-6 months ☐ Over 6 months<br>**Comments:**	

3.8 Metrics on the Approval Process

It would be very helpful to have the following information on processing times for marketing authorisations that have been received and/or determined in the three years 2015, 2016, 2017

3.8.1 Actual approval times (average)

	Time from receipt of application to issue of approval		
**Type**	**2015**	**2016**	**2017**
New Active Substances approved			
Major line extensions approved			
Generics approved			
WHO Pre-qualified generics approved			

PART 4. GOOD REVIEW PRACTICES (GREVP)
BUILDING QUALITY INTO THE REGULATORY PROCESS

Quality in the assessment and registration process is important to regulatory authorities as it ensures consistency, transparency, timeliness and competency in the review processes. Regulatory authorities are continuously developing and implementing a variety of measures to improve and achieve higher quality standards and to meet the expectations of industry and the general public.

The purpose of this section of the questionnaire is to obtain an insight into the strategies, measures and resources that agencies have in place to develop and maintain quality in their review processes.

4.1. General measures used to achieve quality

Please indicate the quality measures currently in place and, where there are none, what, if any, plans there are to introduce such measures in the foreseeable future.

Good Review Practices (GRevP): A code about the process and the documentation of review procedures that aims to standardise and improve the overall documentation and ensure timeliness, predictability, consistency and high quality of reviews and review reports.

4.1.1 *How does your agency define GRevP:* Is it different from the glossary? ☐**YES** ☐**NO**

If different, please define here:

Please outline the key elements that make up GRevP in your agency:

---

*4.1.2 Has the agency **formally or informally** implemented GRevP?*

☐ **YES (Formally)**

☐ **YES (Informally)**

☐ **NO**

If **YES,** please give the title and date of **formal** implementation:

.

4.1.3 How has this been implemented? (Please select the appropriate box(s)):

☐Guidelines ☐ Standard Operating Procedure ☐ GRevP Training Program

☐Other (Please specify):

4.1.4 Are these documents open and available to the public? ☐ **YES** ☐ **NO**

If **YES,** please describe how:

4.1.5 Was the establishment of your GRevP based on other agencies or International standards? ☐**YES** ☐**NO**

If **YES,** please state the name of the agency(ies)/ or internationals standards on which your GRevP has been based:

4.1.6 Are you satisfied with your existing GRevP framework?

☐Satisfied ☐ Could be improved ☐Unsatisfied

If **could be improved** or **unsatisfied,** please select the reason(s) that best describes your situation:

☐ System still evolving

☐ Requires additional training to understand and learn about Good Review Practice
☐ Poor acceptance/utilization by staff

☐ Benefits of implementing GRevP are not apparent so far
☐ Other (please provide details):

4.1.7 If you **do not have** a formal GRevP system in place are there plans to establish this within the next two years? ☐ **YES** ☐ **NO**

Internal Quality Policy: Overall intentions and direction of an organisation related to quality as formally expressed by top management.

4.1.8 Does the agency have an Internal Quality Policy? ☐ **YES** ☐ **NO**

If **NO,** are there plans to establish this within the next two years? ☐ **YES** ☐ **NO**

SOPs (Standard Operating Procedures) are written documents that describe in detail the routine procedures to be followed for a specific operation.

4.1.9 Are there SOPs for the guidance of scientific assessors? ☐ **YES** ☐ **NO**

If **NO,** are there plans to establish SOPs within the next two years? ☐ **YES** ☐ **NO**

4.1.10 Are there SOPs for the advisory committee consulted during the review process? ☐ **YES** ☐ **NO** ☐ **NO COMMITTEE**

If **NO,** are there plans to establish SOPs within the next two years? ☐ **YES** ☐ **NO**

4.1.11 Are SOPs used for any other procedures in the regulatory review process (e.g., validation)? ☐ **YES** ☐ **NO**

If **YES**, please specify:

Assessment Templates set out the content and format of written reports on scientific reviews.

4.1.12 Are there Assessment Templates for reports on the scientific review of an NAS? ☐ **YES** ☐ **NO**

If **NO,** are there plans to establish this within the next two years? ☐ **YES** ☐ **NO**

If **YES,** are these based on another agency's assessment template? ☐ **YES** ☐ **NO**

If **YES,** please specify on which agency was the assessment template based?

4.1.13 Is there an SOP for completing an assessment template? ☐ **YES** ☐ **NO**

4.1.14 Select which elements from the list below are included in your agency assessment template:

☐ Drug Substance
☐ Drug Product
☐ Comments on label
☐ Non-clinical GLP Aspects
☐ Non-clinical Pharmacokinetic
☐ Toxicology
☐ Regulatory background (worldwide status on regulatory agencies)
☐ Other (please specify):
☐ GCP aspects
☐ Clinical Pharmacology (PK & PD)
☐ Clinical Efficacy
☐ Clinical Safety
☐ List of questions for sponsors
☐ Benefit Risk Reduction
☐ Ethnic factors (e.g., consideration of bridging studies)

4.1.15 Would the agency be open to sharing their assessment template or points to consider with CIRS? ☐**YES** ☐**NO**

4.1.16 Do you produce an assessment report (AR) following the review? ☐**YES** ☐**NO**

If **YES:**	
Is there an SOP for completing the AR?	☐ **YES** ☐ **NO**
What language is the AR prepared in?	☐ **LOCAL LANGUAGE**<br>☐ **ENGLISH**
4.1.17 Do you share your AR with other regulatory authorities?	☐ **YES** ☐ **NO**<br>☐ **SOMETIMES**
Do you put your full AR on the website?	☐ **YES** ☐ **NO**<br>☐ **SOMETIMES**
Do you put your abridged AR on the website?	☐ **YES** ☐ **NO**<br>☐ **SOMETIMES**
Do sponsors get a copy of the **full** assessment report?	☐ **YES** ☐ **NO**
Do sponsors have any involvement in the following in relation to AR:	
Preparation of assessment reports	☐ **YES** ☐ **NO**
Comments on the assessment reports	☐ **YES** ☐ **NO**
Translation of assessment reports	☐ **YES** ☐ **NO**
Distribution of assessment reports	☐ **YES** ☐ **NO**
**Peer Review** is an additional evaluation of an original assessment that is carried out by an independent person or committee. Peer review can occur either during assessment of a dossier or at the time of sign-off.	
*4.1.18 Are **external** peer reviews carried out when a NAS is assessed?*	☐ **YES** ☐ **NO**
If **NO,** are there plans to introduce these within the next two years?	☐ **YES** ☐ **NO**
*4.1.19 Are **internal** peer reviews carried out when a NAS is assessed?*	☐ **YES** ☐ **NO**
If **NO,** are there plans to introduce these within the next two years?	☐ **YES** ☐ **NO**
4.1.20 Are there other general procedures in place to monitor the quality of the review process?<br>*What other tools does your agency use to build quality into the assessment process?* (e.g., Internal procedure could include: quality assurance and quality control meeting; stakeholder meeting; channel for grievance; survey of performance from sponsors) Please specify:	

4.2. Quality Management

Reasons for introducing quality measures in the agency

4.2.1 From the following list, please select the ***three*** *most important reasons for the introduction of quality measures:*

- ☐ To be more efficient
- ☐ To minimise errors
- ☐ To ensure consistency
- ☐ To increase transparency
- ☐ To achieve stakeholder satisfaction
- ☐ To improve communications in the agency
- ☐ To improve process predictability
- ☐ To allocate the regulatory resources
- ☐ Other (please specify):

Monitoring to improve quality

4.2.2 Which of the following activities are undertaken by the agency to bring about continuous improvement in the assessment and registration process?

• Reviewing assessors' feedback and taking necessary action	☐ **YES**	☐ **NO**
• Reviewing stakeholders' feedback (e.g. through complaints, meetings or workshops) and taking necessary action	☐ **YES**	☐ **NO**
• Using an internal tracking system to monitor (e.g. consistency, timeliness, efficiency and accuracy)	☐ **YES**	☐ **NO**
• Carrying out internal quality audits (e.g. self-assessments) and using findings to improve the system	☐ **YES**	☐ **NO**
• Having external quality audits by an accredited certification body to improve the system	☐ **YES**	☐ **NO**
• Having a 'post approval' discussion with the sponsor to provide feedback on the quality of the dossier and obtain the company's comments	☐ **YES**	☐ **NO**

Management responsibility for quality

4.2.3 Does the agency have a dedicated department for assessing and/or ensuring quality in the assessment and registration process? ☐ **YES** ☐ **NO**

If **YES**, how many staff are involved?

How often do you assess and/or ensure quality in the assessment and registration process?

☐ **Annually** ☐ **Semi-annually** ☐ **Ad hoc** ☐ **Other (Occasionally)**

To whom does this section report (e.g., the Chief Executive Officer of the agency)?

If **NO**, is the agency thinking of setting up such a department? ☐ **YES** ☐ **NO**

4.3 Quality in the Review and Assessment Process

Improving the quality of applications

4.3.1 Does the agency have official guidelines to assist industry in the registration of medicinal products? ☐ **YES** ☐ **NO**

If **YES**, how are these guidelines made available? (Please indicate all that apply)

☐ Through the agency's website ☐ Through official publications
☐ On request ☐ Through Industry associations
☐ Other, please specify:

What language/s are the guidelines available in:

☐ Local language only ☐ English ☐ Other, please specify:

Improving quality through interactions with applicants

4.3.2 Does the agency provide pre-submission scientific advice to applicants? ☐ **YES** ☐ **NO**

If **YES,** how is the quality of that advice monitored?

4.3.3 Is the applicant given details of technical staff that can be contacted to discuss an application during review? ☐ **YES** ☐ **NO**

4.3.4 Please indicate which of the following best describes the level of contact that companies have with agency staff or outside experts during development and during the agency's assessment:

	Development	*Assessment*
Extensive formal contact (including scheduled meetings)	☐	☐
Extensive informal contact (frequent telephone or email contact)	☐	☐
Some formal contact (possibility of meetings)	☐	☐
Some informal contact (possibility of telephone or email contact)	☐	☐
None, or minimal formal contact (rare occurrences of contact, via letter or fax)	☐	☐
None, or minimal informal contact (rare telephone or email contact)	☐	☐

Please comment on general policy for contact with applicants:

Scientific Committee Procedures

4.3.5 If your review procedure includes obtaining the advice of a scientific committee of internal and/or external experts (as in Section 3.15.1) please complete the following:

Name of the Committee :
Number of Committee members :

How frequently does the Committee meet?

☐ Once a week ☐ Once a month ☐ Other, please specify:

For NAS applications and major line extensions does the Committee review:

☐ All applications ☐ Selected dossiers (Please specify):

Does the Committee review:

☐ The complete dossier ☐ Assessment reports from the reviewers

Shared and Joint reviews with other Regulatory Agencies outside of your country

A ***shared review*** is one where each participating agency takes responsibility for reviewing a separate part of the dossier. A ***joint review*** is one where the whole dossier is reviewed by each agency and the outcome is discussed before a decision is taken.

4.3.6 Is your agency part of any regional alignment initiatives?<br>If **YES**, please specify:<br><br>*4.3.7* Are bilateral/multilateral information sharing agreements in place with other jurisdictions?<br><br>If **YES**, what is the general nature of those agreements?<br><br>*4.3.8 Does your agency conduct shared or joint reviews with other regulatory authorities?*<br>☐ **YES, regularly. Please state which authorities:** ☐ **YES, occasionally. Please state which authorities:**<br>☐ **NO, this has never been undertaken**	☐ **YES** ☐ **NO**<br><br><br>☐ **YES** ☐ **NO**
If **YES**, do you have formal measures in place to ensure consistent quality during the review?<br>If **YES**, please specify:<br><br>If **NO**, do you anticipate undertaking such reviews within the next two years?	☐ **YES** ☐ **NO**<br><br><br>☐ **YES** ☐ **NO**
4.3.9 Have these joint reviews influenced the way in which your agency conducts reviews in general? If so, please comment:	☐ **YES** ☐ **NO**

4.4. Training and continuing education as an element of quality

The following questions relate to training and continuing education of assessors working within the agency, including those employed on a full-time basis and those contracted for specific assessments were necessary.

4.4.1 Do you have a formal training programme for assessors?	☐ **YES** ☐ **NO**
4.4.2 Which of the following methods are used for training assessors?<br>☐ Induction training ☐ External courses<br>☐ On job training ☐ Post-graduate degrees<br>☐ Placements and secondments in other regulatory authorities ☐ Participation in international workshops/ conferences<br>☐ External speakers invited to the agency ☐ In-house courses<br>☐ Other, please specify:	
**Collaboration with other agencies**	
4.4.3 Does your agency seek ***direct*** *assistance of more experienced agencies for development of SOPs and Guidelines?*<br>If **YES,** please give details:	☐ **YES** ☐ **NO**
4.4.4 Does your agency mainly develop SOP, Guidelines etc., based on information published by more experienced agencies:	☐ **YES** ☐ **NO**
4.4.5 Does your agency collaborate with other agencies in the training of assessors?<br>If **YES**, please give details:	☐ **YES** ☐ **NO**
4.4.6 Is training tested in examination situations once completed?	☐ **YES** ☐ **NO**<br>☐ **PARTLY**
4.4.7 Is completion of training courses required for professional advancement?	☐ **YES** ☐ **NO**<br>☐ **PARTLY**

4.5 Transparency of the review process

This section examines ‘transparency’ in terms of the ability and willingness of the agency to assign time and resources to providing information on its activities to both the informed public (which includes health professionals) and industry.

4.5.1 What priority does your agency assign to being open and transparent in relationships with the public, professions and industry?

☐ High priority ☐ Medium priority ☐ Low priority

Please comment:

4.5.2 What are the main drivers for establishing transparency? Please indicate the top ***three*** *incentives for assigning resources to activities that enhance the openness of the regulatory system:*

☐ Political will
☐ Press and media attention
☐ Need to provide assurances on safety safeguards
☐ Public pressure
☐ Need to increase confidence in the system
☐ Better staff morale and performance
☐ Other, please specify:

Transparency to the public

The following questions explore the availability of information to the general public on the performance of regulatory authorities.

4.5.3 Please indicate which of the following information items about the assessment and registration of marketing applications is available to the public:

☐ Approval of products
☐ Approval times
☐ Summary of the grounds on which the approval was granted
☐ Advisory Committee meeting dates
☐ Other, please specify:

4.5.4 How is this information made available?	
☐ Official journal/periodical publication	☐ From an official Internet website
☐ On request	☐ Other, please specify:

Transparency to companies on the application progress

4.5.5 Are companies able to follow the progress of their own applications?	☐ **YES** ☐ **NO**
If **YES,** please indicate the mechanisms available to industry:<br>☐ Electronic access to the status of applications ☐ Telephone contact<br>☐ E-mail contact ☐ Other, please specify	
4.5.6 Are companies given detailed reasons for rejection of an application for registration?	☐ **YES** ☐ **NO**

Facilities for providing information

4.5.7 Is there an electronic system for registering and tracking applications?	☐ **YES** ☐ **NO**
If **YES,** please indicate whether it has the following capabilities:	
• Tracking applications that are under review and identifying the stage in the process	☐ **YES** ☐ **NO**
• Signalling that target review dates have been exceeded	☐ **YES** ☐ **NO**
• Recording the terms of the authorisation once granted	☐ **YES** ☐ **NO**
• Archiving information on applications in a way that can be searched	☐ **YES** ☐ **NO**
If **NO,** are there plans to introduce such a system?	☐ **YES** ☐ **NO**
If so, please give target date for implementation:	

5. QUALITY DECISION-MAKING PROCESSES

Regulatory agencies consider various types of information needed to carry out their assessment of new medicines, but it is not always clear how the decisions, which require human judgment and interpretation, are made around the data. According to the well-established principles of the science of decision making, any organisation that seeks to improve its productivity and consistency should also routinely measure the quality of its decision-making process. These questions aim to uncover the decision-making practices of your agency and focus on the process to approve or reject a New Drug Application.

5.1. Decision-making frameworks

A Framework is a set of principles, guidelines and tools which provide a structured systematic approach to guide decision-makers in selecting, organising, understanding and summarising subjective values and judgments that form the basis of a decision, as well as communicating the evidence relevant to the decision.

5.1.1 Does your agency have a framework in place that forms the basis of the decision to approve or reject a NDA?

☐ Yes ☐ No

If “No”, please answer 5.1.2-5.1.3, if “Yes”, please go to section 5.2 (next page)

5.1.2 Why a framework is not used? (Mark all that apply)

- ☐ Lack of a validated framework
- ☐ Lack of knowledge/training on decision making in general
- ☐ Benefits of a framework not apparent
- ☐ Resource/administrative limitation
- ☐ Others, please specify…………

5.1.3 Are there plans to adopt a framework in the next two years?

☐ Yes ☐ No ☐ Not sure

5.1.4 Which statement best describes the nature of your framework?

- ☐ The framework has been formally defined and codified
- ☐ The framework is informal, by custom and practice (i.e. it has never been clearly agreed but over time has become the process)

5.1.5 In your view, which Quality Decision-Making Practices have been incorporated into your agency’s framework (select one in each row)?

Practice	Practice incorporated	Practice not incorporated	If ‘not incorporated’ tick if you consider it relevant
1. Have a systematic, structured approach	☐	☐	☐
2. Assign clear roles and responsibilities	☐	☐	☐
3. Assign values and relative importance to decision criteria	☐	☐	☐
4. Evaluate both internal and external influences/biases	☐	☐	☐
5. Examine alternative solutions	☐	☐	☐
6. Consider uncertainty	☐	☐	☐
7. Re-evaluate as new information becomes available	☐	☐	☐
8. Perform impact analysis of the decision	☐	☐	☐
9. Ensure transparency and provide a record trail	☐	☐	☐
10. Effectively communicate the basis of the decision	☐	☐	☐

5.2. Decision-making challenges

5.2.1 In your opinion, does your agency actively consider these subjective influences/biases in their decision making?

Subjective influences/biases	Tick if your agency has measures in place to minimise impact of this influence/bias on your agency's decision making	If yes, please describe
**Action-oriented influences** drive us to take action less thoughtfully than we should e.g. Excessive optimism, overconfidence, gut-feeling	☐	
**Interest influences** arise in the presence of conflicting incentives and even purely emotional ones. E.g. misaligned individual incentives and attachments	☐	
**Pattern-recognition influences** lead us to recognize patterns even where there are none e.g. confirmation bias to seek out information that supports a favoured decision	☐	
**Stability influences** create a tendency toward inertia in the presence of uncertainty e.g. preference for the status quo in the absence of pressure to change it	☐	

5.2.2 Does your agency provide training in the area of quality decision making?

☐ Yes ☐ No

Please comment

5.2.3 Are there formal assessments in place to periodically measure the quality of decision-making processes within your agency?

☐ Yes ☐ No

Please comment

5.2.4 Do you think that your agency's decision-making process for approving/rejecting an NDA could be improved?

☐ Yes ☐ No

Please comment, including possible hurdles and solutions

6. CONCLUDING OBSERVATIONS

The purpose of the following two questions is to try to identify the Agency's own perception of its unique positive qualities and the major impediments it faces in carrying out the review of new medicines and making them available to meet patients' needs.

6.1 List three factors that make a major contribution to the effectiveness and efficiency of your agency's review procedures and decision-making processes for NAS applications:
1.
2.
3.
6.2 List three factors that act as barriers to making new medicines available in a timely manner through the regulatory process:
1.
2.
3.

6.3 Are there any important documents related to GRevP that you would like to share with CIRS?

☐ **YES** ☐ **NO**

If yes please list and provide directly to CIRS:

Acknowledgement

Thank you for completing this questionnaire

GLOSSARY AND ABBREVIATIONS

**Additional information**	Additional data or additional analyses of existing data requested from the sponsor by the regulatory agency during the review process.
**Advisory Committee**	An expert committee that advises the regulatory agency of the safety, quality and efficacy of new medicines for human use.
**Approval**	The approval of a drug product by a regulatory agency, signified by the granting of a marketing authorisation, or the issue of a technical approval letter. However, the product may still not be marketable until negotiations for pricing and reimbursement are concluded.
**Clinical summary**	Summary of clinical study data that typically includes biopharmaceutic studies and associated analytical methods, clinical pharmacology studies, clinical efficacy, clinical safety, literature references, and synopses of individual studies. Refers to Module 2.7 in CTD format.
**Common technical document (CTD) format**	Common technical document (CTD) as outlined in the ICH guideline M4 (Organisation of the common technical document for the registration of pharmaceuticals for human use; M4).
**CMC**	Chemistry, manufacturing and controls. All activities conducted to optimize, scale-up and validate the processes and technologies for transfer to manufacture and all QA, QC and CMC support activities (e.g. CMC project management including CMC contribution to project teams). This includes all drug substance R&D i.e. process research and process development, all drug product R&D i.e. formulation development and process development, all analytical work for drug substance R&D and drug product R&D, clinical supplies and CMC's involvement in the compilation of regulatory documentation.
**GCP**	Good Clinical Practice
**Good Review Practices (GRevP)**	A code about the process and the documentation of review procedures that aims to standardise and improve the overall documentation and ensure timeliness, predictability, consistency and high quality of reviews and review reports.
**ICH**	International Conference on Harmonisation
**Internal reviewers**	Internal reviewers are employees of the agency.
**Joint review**	The whole dossier is reviewed by each agency and the outcome is discussed before a decision is taken.
**Marketing Authorisation**	Authorisation issued by a regulatory to launch a drug product on the market.

**Marketing Authorisation Application (MAA)**	Authorisation application submitted to a regulatory agency to launch a drug product on the market to which the application has been submitted.
**Milestone**	A milestone must involve some form of dated written document to which the regulatory agency can refer. In addition, a milestone must be considered by the regulatory agency to be the point at which one event stops and the next one begins so that the times for events are interdependent.
**Major Line Extension**	A major line extension is a modification to an authorised Medicinal Product that is sufficiently great that it cannot be considered to be a simple variation to the original product, but requires a new product authorisation. Such modifications include major new therapeutic indications or new disease states, extension to new patient populations (e.g., paediatrics), a new route of administration or a novel drug delivery system.
**NAS (New Active Substance)**	A new chemical, biological or pharmaceutical active substance includes:<br>a chemical, biological or radiopharmaceutical substance not previously authorised as a medicinal product;<br>an isomer, mixture of isomers, a complex or derivative or salt of a chemical substance not previously authorised as a medicinal product but differing in properties with regard to safety and efficacy from that chemical substance previously authorised;<br>a biological substance previously authorised as a medicinal product, but differing in molecular structure, nature of the source material or manufacturing process;<br>a radiopharmaceutical substance which is radionucleotide, or a ligand not previously authorised as a medicinal product, or the coupling mechanism to link the molecule and the radionucleotide has not been previously authorised.
**Non-clinical summary**	Summary of non-clinical data including: pharmacology, pharmacokinetics and toxicology. Refers to Module 2.6 in CTD format.
**Peer review**	Peer review means an additional evaluation of an original assessment carried out by an independent person or committee. Peer review can occur either during assessment of a dossier, or at sign-off.
**Quality control**	Quality control is operational techniques and activities that are used to fulfil requirements for quality. It involves techniques that monitor a process and eliminate causes of unsatisfactory performance at all stages of the quality cycle.
**Quality policy**	Overall intentions and direction of an organisation related to quality as formally expressed by top management.

**Questions to sponsor**	The process of asking the sponsor for additional data or additional analyses of existing data. The requests are made by the regulatory agency during the review process.
**Scientific assessment**	Review of the dossier in terms of safety, quality and efficacy of data submitted.
**Shared review**	Each agency takes responsibility for assessing a separate part of a dossier.
**Sponsor**	A company, person, organisation or institution that takes responsibility for initiating, managing or financing a clinical study.
**Standard Operating Procedures (SOPs)**	Detailed, written instructions to achieve uniformity of the performance of a specific function
**Validation of a dossier**	The process whereby the agency verifies that all parts of the submitted dossier are present and complete and suitable to be assessed as part of the assessment and registration process.
